# Apremilast for genital erosive lichen planus in women (the AP-GELP Study): study protocol for a randomised placebo-controlled clinical trial

**DOI:** 10.1186/s13063-021-05428-w

**Published:** 2021-07-20

**Authors:** Kristin Helene Skullerud, Petter Gjersvik, Are Hugo Pripp, Erik Qvigstad, Anne Lise Ording Helgesen

**Affiliations:** 1 Norwegian National Advisory Unit on Women’s Health, Oslo University Hospital, Oslo, Norway; 2 Institute of Clinical Medicine, University of Oslo, Oslo, Norway; 3 Oslo Centre for Biostatistics and Epidemiology, Oslo University Hospital, Oslo, Norway; 4 Faculty of Health Sciences, Oslo Metropolitan University, Oslo, Norway; 5 Department of Obstetrics and Gynaecology, Oslo University Hospital, Oslo, Norway; 6 Department of Dermatology, Oslo University Hospital, Oslo, Norway

**Keywords:** Genital erosive lichen planus, Vulval disease, Apremilast, Phosphodiesterase-4 inhibitor, Randomised clinical trial

## Abstract

**Background:**

Genital erosive lichen planus (GELP) is a genital subtype of lichen planus, a chronic autoimmune inflammatory disease of unknown aetiology. In women, GELP is characterised by painful vulvo-vaginal mucosal erosions and scarring, often resulting in poor sexual health and reduced quality of life. Treatment options are limited and often with little effect. Apremilast, a phosphodiesterase 4-inhibitor, has been shown to have a positive effect on psoriasis and other inflammatory skin diseases. We aim to investigate the effect and safety of peroral apremilast in women with GELP in a randomised placebo-controlled double-blinded clinical trial.

**Methods:**

We will recruit 42 adult women with characteristic clinical and/or histological features of moderate-to-severe GELP from a specialised vulva clinic in Oslo, Norway. The patients will be randomised 1:1 to either apremilast 30 mg BID (with an initial dose titration on days 1–6) or a placebo for 24 weeks. The concomitant use of topical corticosteroids will be allowed. The primary end point will be the mean GELP score, a clinical scoring system, at week 24 in the apremilast-treated patients versus the placebo-treated patients. The secondary end points will include the mean GELP score improvement from weeks 0 to 24, patient-reported use of topical steroids, the pain score on a visual analogue scale and the number of patients with GELP score improvements at weeks 16 and 24. The Physician Global Assessment , Patient Global Assessment and selected quality of life and sexual function assessments will be recorded at weeks 0, 16 and 24. The exploratory endpoints include description of immunohistochemical changes before and after apremilast therapy, assessed in vulvar or vaginal biopsies at weeks 0 and 24. Regular follow-ups for possible adverse events will be conducted.

**Discussion:**

The study design is based on experience from studies on apremilast in other inflammatory skin diseases using equivalent apremilast doses for approved indications. The trial may provide evidence for the use of apremilast in women with this burdensome genital dermatosis.

**Trial registration:**

ClinicalTrials.govNCT0365666. Registered on 4 September 2018.

**Supplementary Information:**

The online version contains supplementary material available at 10.1186/s13063-021-05428-w.

## Administrative information

**Table:** 

Title	Apremilast for genital erosive lichen planus in women (The AP-GELP Study): study protocol for a randomised placebo-controlled clinical trial
Trial registration	clinicaltrials.gov NCT0365666
Protocol version	Version 1.7. 21 September 2020
Funding	This is an investigator-initiated trial sponsored by the Oslo University Hospital and the Norwegian National Advisory Unit on Women’s Health and supported by funds from Celgene (from 1 May 2020 Amgen), the manufacturer of apremilast.
Author details	^1^Norwegian National Advisory Unit on Women’s Health, Oslo University Hospital; ^2^Institute of Clinical Medicine, University of Oslo; ^3^Oslo Centre for Biostatistics and Epidemiology, Oslo University Hospital; ^4^Faculty of Health Sciences, Oslo Metropolitan University; ^5^Department of Obstetrics and Gynaecology, Oslo University Hospital; and ^6^Department of Dermatology, Oslo University Hospital; Oslo, Norway
Name and contact information for the trial sponsor	Kirsten Hald, Department of Obstetrics and Gynaecology, Oslo University Hospital E-mail: kirsten.hald@ous-hf.no
Role of sponsor	Oslo University Hospital will pay salaries for investigators and personnel involved in data collection, registration and analysis and will provide basic equipment for study visits. Celgene/Amgen will provide study medication and cover expenses for data collection and monitoring, clinical visits, phone interviews, pharmacy services and immunohistochemical analyses. Sponsor and funders will have no role in the actual collection, management, analysis and interpretation of data, and no ultimate authority in the writing and submission of the report for publication.

## Introduction

Genital erosive lichen planus (GELP) is a genital subtype of lichen planus, which is a chronic autoimmune inflammatory disease [1–3]. In women, GELP is characterised by painful vulvo-vaginal erosions and scarring, and in some cases, this leads to the absorption of the labia and stenosis or total obliteration of the vagina [4]. The diagnosis is based on clinical presentation and/or characteristic histologic findings [5]. The aetiology of GELP is unknown, but autoimmune mechanisms seem to be important [2, 6]. GELP often affects perimenopausal women and is associated with poor sexual health and reduced quality of life [7, 8].

### Limited treatment options

Treatment options for GELP are limited, treatment results are poor, and scientific evidence of treatment efficacy and safety is scarce [2, 3, 9, 10]. Based on clinical experience, the first-line treatment for GELP is the topical application of high-potency corticosteroids, such as clobetasol propionate 0.05%, which usually needs to be used throughout life. Some patients benefit from the addition of topical tacrolimus [11].

Second-line treatments include systemic immunosuppressant or immune-modifying agents such as prednisolone, retinoids, methotrexate, cyclophosphamide, azathioprine, calcineurin inhibitors and mycophenolate mofetil [10, 12, 13]. The use of biologic drugs, including tumour necrosis factor (TNF) inhibitors, has been reported in case reports and case series with inconclusive results.

In 2012, a Cochrane Database systematic review on lichen planus affecting mucosal sites, including GELP, found no evidence for an effect of any therapeutic intervention [14]. In short, the choice of treatment for GELP is based on anecdotal evidence and clinical experience.

In 2015, our group published the first randomised clinical trial involving women with GELP, in which we compared one session of vulvovaginal photodynamic therapy (PDT) with the daily application of high-potent corticosteroids over 6 weeks [15]. For the purpose of the study, we constructed a clinical scoring tool, the GELP score, based on international consensus on the typical features of the disease [5]. The GELP score is based on a severity assessment of the area of genital involvement, erythema, striae and the number of erosions (scored from 0 to 3 by a physician) and patient-reported pain on a visual analogue scale (VAS). GELP scores are calculated for the vulva and the vagina separately (Table 1) [15]. No significant differences were observed between the two groups, but those treated with vulvovaginal PDT reported significantly less use of high-potency topical corticosteroids at the long-term follow-up.

**Table 1:** The GELP scoring system for clinical assessment of genital erosive lichen planus (GELP) in women

Area of involvement	None	0
	< 3 cm^2^	1
	3–6 cm^2^	2
	> 6 cm^2^	3
Intensity of erythema	None	0
	Mild	1
	Moderate	2
	Strong	3
Number of erosions	None	0
	1	1
	2–3	2
	> 3	3
Striae	None	0
	Minimal	1
	Moderate	2
	Extensive	3
Pressure-induced pain (visual analogue scale 0–100 mm)	None (0–9 mm)	0
	10–39 mm	1
	40–69 mm	2
	70–100 mm	3

Vulval and vaginal involvement is assessed separately, resulting in a maximum GELP score of 30

As vulvovaginal PDT requires specialised equipment and many patients with GELP have additional mucosal activity elsewhere, a peroral systemic treatment that is more easily available could be preferable for women with moderate to severe GELP. Apremilast, a phosphodiesterase 4 (PDE4) inhibitor with a documented effect on psoriasis and other inflammatory skin diseases, could be a potential systemic treatment option for GELP, but this has not been studied systematically.

### Apremilast

Apremilast is a small-molecule PDE4 inhibitor [16]. Inhibition of PDE4 results in increased intracellular cyclic AMP, which has effect on several inflammatory pathways, resulting in a decrease in pro-inflammatory cytokines, such as TNF, interferon-γ and interleukin-23, and an increase in anti-inflammatory cytokines [16–18].

Apremilast has been shown to reduce the severity of inflammatory skin diseases, such as psoriasis [19–23], and has been studied in atopic dermatitis, albeit with less success [24]. The effect of apremilast is also being investigated for other autoimmune skin diseases [25]. Apremilast has been found to be effective in the treatment of Behçet’s disease, which has oral and genital ulcers as key features [26, 27], as well as in aphthous stomatitis [28]. In an open-label study with 10 patients, apremilast was reported to be efficacious in the treatment of cutaneous lichen planus [29]. Furthermore, some case reports have indicated an effect in oral lichen planus [30, 31] and lichen planus-associated stenotic oesophagitis [32].

Apremilast has a favourable safety profile and generally tolerable side effects [33], which is essential in the treatment of chronic diseases requiring continuous treatment. Common adverse effects include short-term nausea, diarrhoea, headache, and, less commonly, moderate weight loss and upper respiratory tract infections [20, 21]. In the placebo-controlled ESTEEM 1 and 2 trials, depression was reported by 1.4% of the 832 apremilast-treated patients versus 0.5% of the 418 placebo-treated patients [22].

As of March 2020, it is estimated that more than 480 000 patients have been treated with apremilast [34]. Apremilast is approved for use in several countries in all regions across the world and is accepted for reimbursement for psoriasis in most EU countries. In Norway, apremilast is currently approved for use in the treatment of chronic plaque psoriasis and psoriatic arthritis. With the increasing evidence of an effect against several forms of cutaneous and mucosal inflammatory diseases, apremilast may represent a potential new treatment for GELP in women.

### Rationale and aims of the study

GELP is a burdensome disease with few and poor treatments options. The main objective of this study is to investigate the effect and safety of apremilast as a systemic treatment in women with moderate-to-severe GELP. We will perform a randomised, placebo-controlled, double-blinded clinical trial.

We will also assess quality of life and sexual function before and during apremilast treatment and describe possible immune histochemical changes and the expression of selected cytokines in GELP lesions after treatment with apremilast.

## Materials and methods

### Study setting

The Vulva Clinic at Oslo University Hospital, Oslo, Norway, is a national tertiary referral centre for women with chronic vulval diseases [4]. Two gynaecologists and two dermatologists are employed on a part-time basis, and collaborate with other health care professionals. In 2018, more than 360 consultations were carried out, and approximately half of the patients resided in the southeast region of Norway. By 2019, around 200 patients with genital lichen planus had been registered in the clinic’s data base. The study participants will mainly be recruited from the Vulva Clinic in Oslo, but possibly also from specialists outside Oslo University Hospital.

### Patient recruitment

Forty-two patients will be recruited according to the following inclusion criteria

- Female
- > 18 years of age
- Moderate-to-severe GELP with the diagnosis based on characteristic clinical and/or histological features.

To achieve adequate participant enrolment, we will actively contact other vulva clinics in Norway, as well as dermatologists and gynaecologists in private practice. Information about the trial is accessible on the national health services website.

### Eligibility criteria

Severity of GELP will be recorded using the GELP score, which was used by our group in a previous study [15] and is based on the typical clinical features and pain (Table 1). For the purpose of inclusion, the minimum GELP score will be 5/30 in the vagina and/or vulva (scored separately), of which erythema and pain ≥ 1 will be mandatory. Sexually active women of childbearing potential must agree to use highly effective contraception during the treatment period and 28 days following the last dose of apremilast or placebo.

### Exclusion criteria

The study exclusion criteria are as follows:

- Patients receiving other systemic immune-modulating therapy
- The concomitant use of strong CYP3A4 enzyme inducers
- Inadequate birth control, pregnancy and/or breast-feeding
- Depression and/or suicidal ideation
- Patients with severe renal impairment
- Patients with active tuberculosis, serious infections or cancer
- Unexplained and clinically significant weight loss in underweight patients
- Hypersensitivity to the active substance(s) or to any of the excipients
- Hereditary problems of galactose intolerance, lactase deficiency or glucose-galactose malabsorption
- Participation in another trial that could affect the current study (or there should be a minimum of 90 days between participation in this study and another intervention trial)

Signed informed consent will be obtained from all the participating women by one of the investigators and documented in accordance with international, national and local regulations. Informed consent will cover the use of the participants´ data and biological specimens.

### Randomisation and blinding

The randomisation of patients into one of two arms, apremilast or placebo, will occur at the baseline visit, after all inclusion criteria and no exclusion criteria are fulfilled and the patient is included in the study.

The study site will be provided with batches of treatment kits from Celgene (from 1 May 2020 Amgen) on a regular basis. A specific kit number will identify each treatment kit. Celgene/Amgen is responsible for labelling the kits and provide an unblinded data manager with code lists for each batch containing information about the kit numbers and its associated treatment. No other personnel involved in the study will have access to these code lists. The unblinded data manager will generate the drug allocation lists which will be uploaded to the clinical data management system Viedoc 4. The investigator(s) will receive the patient’s kit number through the electronic case report form (eCRF) system. A treatment kit with the corresponding number will then be given to the patient.

The allocation-sequence will be generated by a person not involved in the trial and done by permuted block randomisation with unequal random block sizes and a 1:1 ratio of allocation (i.e. patients will be allocated to the treatments with equal probabilities) using appropriate statistical software. The details will be provided in a separate document unavailable to the study personnel. The allocation list will be stored at the Clinical Trial Unit at Oslo University Hospital and will not be available to the investigators.

Both the patients and the investigators will be blinded for the allocation for the full treatment period. Unblinding of the treatment allocation will be allowed only if the safety and well-being of the patient makes this necessary. This decision can only be made by one of the investigators.

### Intervention

Apremilast will be tested against a placebo, as no proven effective systemic treatment for GELP exists. All the participants will be allowed to use locally applied high-potency corticosteroids according to their symptoms and will record the frequency of such use weekly. With this add-on study design, the participants in the placebo group will not be subject to the risk of serious or irreversible harm as a result of not receiving the best proven treatment.

Women randomised to the apremilast group will receive study medication with an initial standard titration of dose for days 1–6, followed by a standard dose of 30 mg apremilast BID. Those randomised to the placebo group will receive tablet blister cards identical in appearance to those given to the apremilast group. The apremilast 30 mg and placebo tablets will be administered orally, twice daily (morning and evening), approximately 12 h apart for 24 weeks. To reduce the risk of gastrointestinal symptoms, the dose titration on days 1–6 will be administered according to the following schedule:

- Day 1: 10 mg in the morning
- Day 2: 10 mg in the morning and 10 mg in the evening
- Day 3: 10 mg in the morning and 20 mg in the evening
- Day 4: 20 mg in the morning and 20 mg in the evening
- Day 5: 20 mg in the morning and 30 mg in the evening
- From day 6: 30 mg twice daily

The participants will not be allowed to reduce the apremilast (or placebo) dose. Participants will record any interruptions of dosage in the patient’s diary weekly and will be asked about adherence at each study visit and via telephone consultations. When a patient returns for the next visit, she will be required to bring back blister cards, and any unused medication will be registered.

During the study period, the participants will be allowed to use potent topical corticosteroids (clobetasol propionate or similar) in the genital area as needed. As diarrhoea may cause irritation of the genital area during the first weeks of treatment, patients will be given advice on hygiene measures and use of moisturizing and barrier creams at initiation of treatment. Prohibited concomitant medications during the trial are listed in the exclusion criteria. Other systemic medications taken by the patient, including vitamins, herbal preparation and other over-the-counter drugs, will be recorded in the patient’s file and eCRF.

The patients will be allowed to withdraw from the study or discontinue treatment at any time without having to state their reason(s) for doing so. Patients may be discontinued from the treatment by the principal investigator due to adverse effects, pregnancy or incorrect enrolment. All the patients will be offered clinical consultations a maximum 14 days following withdrawal. At the end of study, i.e. at week 24 or earlier if the patient withdraws from the study, the patients will be given an appointment with one of the investigators after 3–6 months for regular clinical follow-up.

Vulvar or vaginal biopsies will be taken on a voluntary basis at weeks 0 and 24; this will be done only in women who have consented to the procedure.

### Outcomes

The primary and secondary end points were selected to reflect efficacy that is potentially relevant for patients in clinical practice.

The primary endpoint is the mean GELP score, as used by our group in a previous study and described above, at week 24 in the apremilast-treated patients versus the placebo-treated patients (Table 1).

The secondary endpoints are:

- Mean GELP score improvement from week 0 to week 24 in all patients
- Weekly use of topical steroid, as recorded in the patients’ weekly diaries
- Weekly VAS pain score, as recorded in patients’ diaries
- Number of patients with GELP score improvement at weeks 16 and 24
- Separate GELP score assessments: area of involvement (in cm^2^), number of erosions, erythema, striae and pain (VAS) at weeks 4, 16 and 24
- Physician Global Assessment (PGA) [35] at weeks 0, 16 and 24
- Patient Global Assessment (PtGA) [35] at weeks 0, 16 and 24
- Dermatology Life Quality Index (DLQI) [36] and General Health Questionnaire-28 (GHQ-28) [37] at weeks 0, 16 and 24
- Female Sexual Distress Scale (FSDS-R) [38] at weeks 0, 16 and 24

The PGA and PtGA, which will be used by the investigator and the patient, respectively, are five-point disease severity scoring systems used to assess disease severity as clear, almost clear, mild, moderate or severe. The frequency of the topical application of high-potency corticosteroid will be recorded as the number of days of use per week.

The exploratory endpoints are description of immunohistochemical changes and expression of selected cytokines in vulvar or vaginal biopsies at weeks 0 and 24, description of extragenital lichen planus at weeks 0 and 24, clinical photos at weeks 0 and 24, and adverse events.

### Participant timeline

**Fig. 1:**
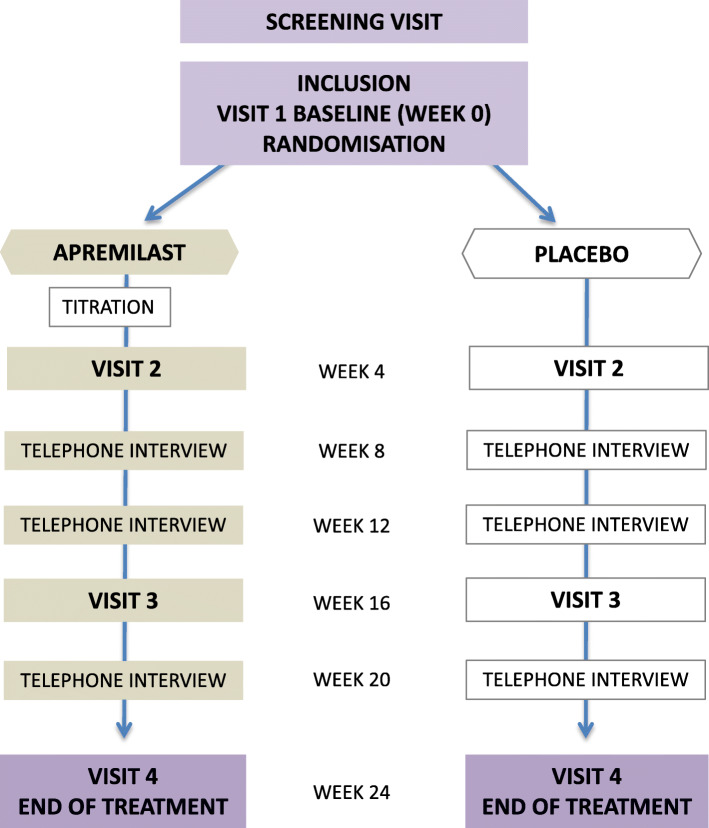
Flow chart. Flow chart for the randomised placebo-controlled clinical trial on apremilast for genital erosive lichen planus in women (the AP-GELP Study)

Screening will be performed no more than 90 days prior to baseline visit (Fig. 1 and Table 2). The patients will receive information on study participation at the screening visits. At baseline visit, patients who have qualified to participate in the study based on the inclusion and exclusion criteria, and who have signed the informed consent form, will be randomised to receive either apremilast or the placebo. Screening and baseline visits may be on the same day, but the patients will preferably have at least 24 h between screening and baseline visits to allow adequate time for patient information and informed consent to be obtained.

**Table 2:** Schedule of enrolment, interventions and assessments

Procedure	Participant contact								
	Screening	Baseline inclusion	Visit 2	Telephone interview	Telephone interview	Visit 3	Telephone interview	End of treatment visit	Withdrawal visit
Week ± 7 days	**Max − 90 days**	**0**	**4**	**8**	**12**	**16**	**20**	**24**	**Max 14 days after**
Informed consent	x								
Eligibility assessment	x								
Randomisation		x							
Study treatment		x	x	x	x	x	x	x	
Compliance			x	x	x	x	x	x	x
Medical history	x	x	x			x		x	x
Safety evaluation	x	x	x	x	x	x	x	x	x
Clinical examination	x	x	x			x		x	x
Weight	x	x	x			x		x	x
PGA		x				x		x	x
PtGA		x				x		x	x
DLQI		x				x		x	x
FSDS-R		x				x		x	x
GHQ-28	x	x	x			x		x	x
GELP score	x	x	x			x		x	x
Vulval/vaginal biopsy		x						x	
HCG^1^	x	x	x	x	x	x	x	x	x
GFR^1^	x								
IGRA^1^	x								
Collect patient diary						x		x	
Clinical photo		x						x	

^1^Blood or urine (HCG) sample

Clinical examinations will be performed by one of the investigators at weeks 0, 4, 16 and 24. The examinations will include a clinical assessment of GELP score, PGA score and clinical photos, as well as an inspection of the oral cavity and skin, cardiac and pulmonary auscultation, and blood pressure measurements. The patients will complete the validated patient questionnaires (PtGA, DLQI, GHQ-28 and FSDS-R).

All the patients will use an electronic diary (ViedocMe) or an optional paper version for weekly registration of the use of topical corticosteroids (number of days per week), genital pain assessment (VAS scale 0–100) and adverse events. Paper diaries will be collected at weeks 16 and 24. At weeks 8, 12 and 20, the participants will be contacted by phone by a study nurse and asked about possible side effects and compliance, and will be encouraged to fill in the diary log. The interview will specifically focus on any psychiatric symptoms and, for women with child-bearing potential, the results of a home pregnancy test.

In addition to the regular clinical and telephone-based contacts, all the trial participants will have the opportunity to contact the trial physicians by phone and/or e-mail, both during and after the trial period.

### Drop-out criteria

Participants may be discontinued from the study treatment and/ or the assessments at any time due to voluntary discontinuation by the patient, patient loss to follow-up, safety reasons, pregnancy or incorrect enrolment.

### Data management

Using Viedoc, the investigator staff will enter data into the eCRF in accordance with the protocol. The investigator is responsible for assuring that the data entered into the eCRF are complete, accurate, and that entries are performed according to schedule. Data will be validated as defined in a data validation plan, including validity, consistency and customised checks. Participants will record any dosage interruptions in the weekly patient’s diary and will be asked about adherence at each study visit and telephone consultation, to promote participant retention and complete the follow-up.

The investigators will arrange for the secure retention of patient identification, code list and eCRFs, which will be stored for 15 years after the study closure, inaccessible to unauthorised personnel. The biopsies will be stored in accordance with technical and regulatory requirements.

### Statistics

In the randomised clinical trial on the effect of vulvovaginal PDT, mean GELP score was reduced from 11 (standard deviation (SD) 4.5) at baseline to 7 (SD 4.2) after 24 weeks [15]. We assume that a similar mean difference and SD in the GELP scores at week 24 between the apremilast-treated and placebo-treated patients will be a likely clinically important difference. To obtain 80% statistical power from an independent samples t test with a 5% significance level, mean difference of 4 and standard deviations in the two groups of 4.5 and 4.2, 20 subjects will be required in each group. Based on this sample size calculation and to account for a small drop-out rate, we decided to include 42 patients.

The demographic and baseline characteristics will be presented using the mean, SD, number of observations and percentages as appropriate.

The treatment effect will be analysed using both an intention-to-treat design (main analysis) and a per-protocol design. The main statistical analysis will be performed when the planned number of patients have either finalised their week 24 assessments or have withdrawn from the trial and when all the data have been registered, verified and validated according to the data management plan. A separate statistical analysis plan will provide further details regarding the statistical analyses.

The primary endpoint will be assessed using an ANCOVA model adjusting for the GELP score at baseline, with a 5% significance level. The results will be reported as the mean difference between the apremilast-treated patients and the placebo-treated patients with a 95% confidence interval and a *p*-value as estimated in the ANCOVA model.

The continuous secondary outcomes at the 4-, 16- and 24-week follow-ups will be assessed using the same statistical principle as for the primary outcome (i.e. an ANCOVA model adjusting for the measured outcome at baseline). The categorical outcomes will be assessed with Pearson chi-square tests or logistic regression models as appropriate. In addition, statistical analyses will be conducted using mixed models for repeated measurements.

Interim and subgroup analyses will not be performed. If non-adherence and/or missing data are regarded as having a significant effect on the conclusions of the study, sensitivity analyses with methods for handling missing data will be performed. Such methods may include complete case analyses, last observation carried forward, worst case/best case imputation and multiple imputation techniques. Statistical analysis with mixed models for repeated measurements will also be conducted to obtain robust estimates in case of missing data.

### Adverse events

The investigator is responsible for the detection and documentation of any adverse events, including serious adverse events. The study personnel will communicate with the participants at least once a month at visits or via phone calls. Participants will be able to contact the study personnel by phone or e-mail 24 h a day throughout the treatment period.

The patients will be instructed to contact the investigator immediately if they experience signs or symptoms they perceive as serious. At the time of inclusion in the study, a letter with information on the study and potential adverse effects will be sent to the patient’s primary care physician. The participants will be encouraged to inform their family about the risk of changes in behaviour or mood and any suicidal thoughts.

Any adverse events will be described in the eCRF according to duration, severity/intensity, causality related to study treatment, the action taken with study treatment and outcome of the adverse event. During the course of the study all adverse events will be proactively followed up for each patient.

### Pregnancy

A pregnancy occurring while the participant is on treatment in the study or within 28 days after the last dose is considered an immediately reportable event, and the study treatment is to be discontinued immediately. The pregnant woman will be referred to appropriate healthcare professionals for further evaluation.

### Data monitoring

The study will be monitored on a regular basis by the Clinical Trial Unit, who will check the following:

• Informed consent process

• Reporting of adverse events and all other safety data

• Adherence to protocol

• Maintenance of required regulatory documents

• Study supply accountability

• Data completion in the eCRFs including source data verification

A safety committee has been established to assess safety concerns. An external trial steering committee was not deemed necessary due to the small study size and the study being performed at only one centre.

### Protocol amendments

Possible protocol modifications will be reported to the Regional Ethical Committee, the Norwegian Medicines Agency, the sponsor, Celgene/Amgen and clinicaltrials.gov according to guidelines. Relevant protocol amendments will also be communicated to trial participants

### Access to data

The full protocol is available as an additional file. The data sets and statistical codes may be made available to external researchers on reasonable request. No access will be given to participant-level data.

### Dissemination plans

Upon study completion and analysis, the results will be submitted for publication in a peer-reviewed scientific medical journal and/or posted in a publicly accessible database of clinical studies. The results will also be submitted to the relevant authorities according to European Union and national regulations, as well as to the participating patients. The investigators will have the right to publish the report regardless of outcomes.

## Discussion

To our knowledge, this is the first randomised controlled trial using peroral apremilast in women with GELP. The protocol has been subject to external reviews by the Norwegian Medicine Agency and the South-East Regional Committee on Medical and Health Research Ethics in Norway and amended according to their advice and requirements.

Many women with GELP are highly motivated to be included in clinical trials. Recently, however, a multicentre randomised controlled pilot study on systemic therapy for vulval erosive lichen planus (the ‘hELP’ trial) [39] was stopped before reaching its recruitment target of 40 patients [40]. Of the 22 patients who entered the four-armed trial, 10 patients either did not start the treatment, stopped the treatment or were lost to follow-up. This confirms some of the challenges faced when running a randomised clinical trial in patients with a relatively rare disease. Nevertheless, we hope that our trial may provide evidence regarding the use of apremilast in women with this burdensome genital disease, which tends to be neglected by many researchers and physicians. The study may also provide insights into the quality of life and sexual health of women with GELP and tissue samples for use in immunological studies on the pathogenesis of GELP.

## Trial status

The trial protocol, version 1.6, was finalised on 16 October, 2019, and updated on 21 September, 2020. The first patient was included on 24 September, 2019, and the last patient is scheduled to complete the trial by the end of 2021. Due to the COVID-19 pandemic, inclusion was paused between March and August 2020.

## Supplementary Information

**Additional file 1.** AP-GELP Study Protocol, version 1.7.

**Additional file 2.** SPIRIT checklist.

## Data Availability

The data sets may be made available by the corresponding author on reasonable request.

## Abbreviations

CRF: Case report form
eCRF: Electronic case report form
EMA: European Medicine Agency
FSDS: Female Sexual Distress Scale
GELP: Genital erosive lichen planus
GHQ-28: General Health Questionnaire-28
PDE4: Phosphodiesterase 4
PDT: Photodynamic treatment
PGA: Physician Global Assessment
PtGA: Patient Global Assessment
SAP: Statistical analysis plan
TNF: Tumour necrosis factor
VAS: Visual analogue scale

## References

[1] Lewis FM, Bogliatto F (2013). Erosive vulval lichen planus--a diagnosis not to be missed: a clinical review. Eur J Obstet Gynecol Reprod Biol..

[2] Zendell K (2015). Genital lichen planus: update on diagnosis and treatment. Semin Cutan Med Surg..

[3] Mauskar M (2017). Erosive lichen planus. Obst Gynecol Clin North Am..

[4] Helgesen AL, Gjersvik P, Jebsen P, Kirschner R, Tanbo T (2010). Vaginal involvement in genital erosive lichen planus. Acta Obstet Gynecol Scand..

[5] Simpson RC, Thomas KS, Leighton P, Murphy R (2013). Diagnostic criteria for erosive lichen planus affecting the vulva: an international electronic-Delphi consensus exercise. Br J Dermatol..

[6] Cooper SM, Dean D, Allen J, Kirtschig G, Wojnarowska F (2005). Erosive lichen planus of the vulva: weak circulating basement membrane zone antibodies are present. Clin Exp Dermatol..

[7] Lundqvist EN, Wahlin YB, Bergdahl M, Bergdahl J (2006). Psychological health in patients with genital and oral erosive lichen planus. J Eur Acad Dermatol Venereol..

[8] Cheng H, Oakley A, Conaglen JV, Conaglen HM (2017). Quality of life and sexual distress in women with erosive vulvovaginal lichen planus. J Low Genit Tract Dis..

[9] Moyal-Barracco M, Edwards L (2004). Diagnosis and therapy of anogenital lichen planus. Dermatol Ther..

[10] Ho JK, Hantash BM (2012). Systematic review of current systemic treatment options for erosive lichen planus. Exp Rev Dermatol..

[11] Kirtschig G, van der Meulen AJ, Ion Lipan JW, Stoof TJ (2002). Successful treatment of erosive vulvovaginal lichen planus with topical tacrolimus. Br J Dermatol..

[12] Deen K, McMeniman E (2015). Mycophenolate mofetil in erosive genital lichen planus: a case and review of the literature. J Dermatol.

[13] Yeo L, Ormerod AD (2016). Oral tacrolimus: a treatment option for recalcitrant erosive lichen planus. Clin Exp Dermatol.

[14] Cheng S, Kirtschig G, Cooper S, Thornhill M, Leonardi-Bee J, Murphy R (2012). Interventions for erosive lichen planus affecting mucosal sites. Cochrane Database Syst Rev..

[15] Helgesen AL, Warloe T, Pripp AH, Kirschner R, Peng Q, Tanbo T (2015). Vulvovaginal photodynamic therapy vs. topical corticosteroids in genital erosive lichen planus: a randomized controlled trial. Br J Dermatol..

[16] Kumar N, Goldminz AM, Kim N, Gottlieb AB (2013). Phosphodiesterase 4-targeted treatments for autoimmune diseases. BMC Med..

[17] Perez-Aso M, Montesinos MC, Mediero A, Wilder T, Schafer PH, Cronstein B (2015). Apremilast, a novel phosphodiesterase 4 (PDE4) inhibitor, regulates inflammation through multiple cAMP downstream effectors. Arthritis Res Ther..

[18] Schafer PH, Truzzi F, Parton A, Wu L, Kosek J, Zhang L-H, Horan G, Saltari A, Quadri M, Lotti R, Marconi A, Pincelli C (2016). Phosphodiesterase 4 in inflammatory diseases: Effects of apremilast in psoriatic blood and in dermal myofibroblasts through the PDE4/CD271 complex. Cell Signal..

[19] Strand V, Fiorentino D, Hu C, Day RM, Stevens RM, Papp KA (2013). Improvements in patient-reported outcomes with apremilast, an oral phosphodiesterase 4 inhibitor, in the treatment of moderate to severe psoriasis: results from a phase IIb randomized, controlled study. Health Qual Life Outcomes..

[20] Papp K, Reich K, Leonardi CL, Kircik L, Chimenti S, Langley RG (2015). Apremilast, an oral phosphodiesterase 4 (PDE4) inhibitor, in patients with moderate to severe plaque psoriasis: results of a phase III, randomized, controlled trial (Efficacy and Safety Trial Evaluating the Effects of Apremilast in Psoriasis [ESTEEM] 1). J Am Acad Dermatol..

[21] Paul C, Cather J, Gooderham M, Poulin Y, Mrowietz U, Ferrandiz C, Crowley J, Hu C, Stevens RM, Shah K, Day RM, Girolomoni G, Gottlieb AB (2015). Efficacy and safety of apremilast, an oral phosphodiesterase 4 inhibitor, in patients with moderate-to-severe plaque psoriasis over 52 weeks: a phase III, randomized controlled trial (ESTEEM 2). Br J Dermatol..

[22] Crowley J, Thaci D, Joly P, Peris K, Papp KA, Goncalves J (2017). Long-term safety and tolerability of apremilast in patients with psoriasis: Pooled safety analysis for >/=156 weeks from 2 phase 3, randomized, controlled trials (ESTEEM 1 and 2). J Am Acad Dermatol..

[23] Keating GM (2017). Apremilast: a review in psoriasis and psoriatic arthritis. Drugs..

[24] Samrao A, Berry TM, Goreshi R, Simpson EL (2012). A pilot study of an oral phosphodiesterase inhibitor (apremilast) for atopic dermatitis in adults. Arch Dermatol..

[25] Maloney NJ, Zhao J, Tegtmeyer K, Lee EY, Cheng K (2020). Off-label studies on apremilast in dermatology: a review. J Dermatol Treat.

[26] Hatemi G, Melikoglu M, Tunc R, Korkmaz C, Ozturk BT, Mat C (2015). Apremilast for Behçet’s syndrome--a phase 2, placebo-controlled study. N Engl J Med..

[27] Hatemi G, Mahr A, Ishigatsubo Y, Song Y, Takeno M, Kim D (2019). Trial of apremilast for oral ulcers in Behçet’s syndrome. N Engl J Med..

[28] Kolios AGA, Yawalkar N, Feusi A, Kündig T, Boyman O, Nilsson J (2019). Apremilast in treatment-refractory recurrent aphthous stomatitis. N Engl J Med..

[29] Paul J, Foss CE, Hirano SA, Cunningham TD, Pariser DM (2013). An open-label pilot study of apremilast for the treatment of moderate to severe lichen planus: a case series. J Am Acad Dermatol..

[30] Bettencourt M (2016). Oral lichen planus treated with apremilast. J Drugs Dermatol..

[31] AbuHilal M, Walsh S, Shear N (2016). Treatment of recalcitrant erosive oral lichen planus and desquamative gingivitis with oral apremilast. J Dermatol Case Rep..

[32] Hafner J, Gubler C, Kaufmann K, Nobbe S, Navarini AA, French LE (2016). Apremilast Is effective in lichen planus mucosae-associated stenotic esophagitis. Case Rep Dermatol..

[33] Nast A, Spuls PI, van der Kraaij G, Gisondi P, Paul C, Saiag P (2017). European S3-Guideline on the systemic treatment of psoriasis vulgaris - update apremilast and secukinumab - EDF in cooperation with EADV and IPC. J Eur Acad Dermatol Venereol..

[34] Apremilast Investigators Brochure 23.0. Section 6.3. Amgen: 2020.

[35] Pascoe VL, Enamandram M, Corey KC, Cheng CE, Javorsky EJ, Sung SM, Donahue KR, Kimball AB (2015). Using the Physician Global Assessment in a clinical setting to measure and track patient outcomes. JAMA Dermatol..

[36] Finlay AY, Khan GK (1994). Dermatology Life Quality Index (DLQI)--a simple practical measure for routine clinical use. Clin Exp Dermatol..

[37] Goldberg D, Hillier V (1979). A scaled version of the General Health Questionnaire. Psychol Med..

[38] Derogatis LR, Rosen R, Leiblum S, Burnett A, Heiman J (2002). The Female Sexual Distress Scale (FSDS): initial validation of a standardized scale for assessment of sexually related personal distress in women. J Sex Marital Ther..

[39] Simpson RC, Murphy R, Bratton DJ, Sydes MR, Wilkes S, Nankervis H (2016). Systemic therapy for vulval erosive lichen planus (the ‘hELP’ trial): study protocol for a randomised controlled trial. Trials.

[40] Simpson RC, Murphy R, Bratton DJ, Sydes MR, Wilkes S, Nankervis H, Dowey S, Bell H, Cruickshank M, Gibbon K, Green CM, Wong C, Owen CM, London K, Haque S, Thomas KS (2018). Help for future research: lessons learned in trial design, recruitment, and delivery from the ‘hELP’ study. J Low Genit Tract Dis.

