# Supplementary material for: Apremilast for genital erosive lichen planus in women (the AP-GELP Study): study protocol for a randomised placebo-controlled clinical trial
Source: Trials. 2021 Jul 20;22:469. doi: 10.1186/s13063-021-05428-w (PMC8290211; doi:10.1186/s13063-021-05428-w)
Supplement: Supplementary file 1 — Additional file 1. AP-GELP Study Protocol, version 1.7. [file 13063_2021_5428_MOESM1_ESM.pdf]

THE AP-GELP STUDY: A RANDOMIZED, PLACEBO-CONTROLLED CLINICAL TRIAL ON THE EFFECTS OF PHOSPHODIESTERASE 4-INHIBITOR APREMILAST IN FEMALE GENITAL EROSIVE LICHEN PLANUS

Protocol Identification Number: APGELP

EudraCT Number: 2017-004116-19

Clinical trial registration number: NCT03656666

Regional committee for medical and health research ethics number: 2018/1841

Sponsor: **Oslo University Hospital HF**

Norwegian National Advisory Unit on Women's Health

Rikshospitalet

P.O. Box 4950 Nydalen

NO-0424 Oslo, Norway

Tel: +47 23072683

Protocol version no. 1.7

21 September 2020

CONTACT DETAILS

**Sponsor:**	**Oslo University Hospital HF**<br>**Norwegian National Advisory Unit on Women's Health**<br>P.O. box 4950 Nydalen<br>0424 Oslo<br>Tel: +47 23072683
**Principal Investigator:**	**Anne Lise Ording Helgesen, MD PhD**<br>Dept. of Dermatology, Oslo University Hospital HF, Rikshospitalet<br>P.O. Box 4950 Nydalen<br>0424 Oslo<br>Tel: +47 98634403<br>Email: ahelgese@ous-hf.no
**Participating Department:**	**Dept. of Obstetrics and Gynaecology, Oslo University Hospital HF, Ullevål Hospital**<br>P.O. Box 4956 Nydalen<br>0424 Oslo<br>Tel: 02770/ +47 915 02770<br>Email: tone.walstad@ous-hf.no
**Participating Department:**	**Department of Rheumatology, Dermatology and Infectious diseases**<br>P.O. Box 4763 Nydalen, 0506 Oslo<br>Tel: +47 23075840
**Monitor:**	**Clinical Trial Unit**<br>**Oslo University Hospital HF**<br>Sogn Arena, Klaus Torgårds vei 3, 0372 Oslo<br>Email: oushfpbctu@ous-hf.no

SIGNATURE PAGE

Title	The AP-GELP Study: A randomized, placebo-controlled clinical trial on the effects of phosphodiesterase 4-inhibitor apremilast in female genital erosive lichen planus
Protocol ID no:	APGELP
EudraCT no:	2017-004116-19

I hereby declare that I will conduct the study in compliance with the protocol, ICH GCP and the applicable regulatory requirements:

Name	Title	Role	Signature	Date
Kirsten Hald	Head of department, PhD	Sponsor's representative		
Anne Lise Ording Helgesen	MD PhD	Principal Investigator		
Kristin Helene Skullerud	MD	Investigator/ PhD student		
Erik Qvigstad	Professor, MD, PhD	Collaborator		
Petter Gjersvik	Professor, MD, PhD	Collaborator		
Are Hugo Pripp	PhD	Statistician		

PROTOCOL SYNOPSIS

The AP-GELP Study: A randomized, placebo-controlled clinical trial on the effects of phosphodiesterase 4-inhibitor apremilast in female genital erosive lichen planus

**Sponsor**	Oslo University Hospital
**Phase and study type/design**	Phase II, interventional placebo-controlled trial.<br><br>Patients are randomized 1:1 to either apremilast 30 mg BID (interventional drug) or placebo for 24 weeks. The study is double-blinded for the randomization throughout the treatment period.
**Investigational Medical Products (IMP)**	• Apremilast 10/20/30 mg tablets<br>• Placebo tablets
**Center:**	Oslo University Hospital
**Study Period:**	Date of first patient enrolled: 24.09.2019<br><br>Anticipated recruitment period: 01.03.2019 – 01.07.2021<br><br>Estimated date of last patient completed: 31.12.2021
**Treatment Duration:**	Expected treatment duration per patient: 24 weeks<br>End of trial is defined as the last visit of the last patient
**Objectives**	Main study objective<br>• To assess the effect of apremilast treatment on GELP<br><br>Secondary objectives<br>• To assess quality of life in women with GELP before and during apremilast treatment<br>• To assess sexual function in women with GELP before and during apremilast treatment<br><br>Main exploratory objective<br>• To describe immune histochemical changes and expression of selected cytokines in GELP lesions after treatment with apremilast

Endpoints:

Primary endpoint:

- Mean GELP score at week 24 in apremilast-treated patients versus placebo-treated patients

Secondary endpoints:

- Mean GELP score improvement from week 0 to week 24 in all patients
- Weekly use of topical steroid, collected from patient diary
- Weekly VAS pain score, collected from patient diary
- Number of patients with GELP score improvement at week 16 and 24
- Separate GELP score assessments: Area of involvement (in cm^2), number of erosions, erythema, striae and pain (VAS) at week 4, 16 and 24
- Physician Global Assessment (PGA) and Patient Global Assessment (PtGA) scores at week 0, 16 and 24
- Selected QoL measures (DLQI, GHQ-28) at week 0, 16 and 24
- Sexual function assessments (FSDS-R) at week 0, 16 and 24

Exploratory endpoints:

- Description of immune histochemical changes and expression of selected cytokines before and after apremilast therapy, assessed in vulvar or vaginal biopsies at week 0 and 24
- Description of extragenital lichen planus at week 0, 16 and 24
- Clinical photos taken at week 0 and week 24
- Adverse events

Main Inclusion Criteria:

- >18 years of age
- Female
- Moderate to severe GELP at inclusion with the diagnosis based on characteristic clinical and/or histological features. Minimum GELP score 5/30 in vagina and/or vulva (scored separately), of which erythema and pain ≥1 are mandatory
- Informed consent from the patient to the protocol and clinical procedures.

Main Exclusion Criteria

- Patients receiving other systemic immune modulating therapy

- Concomitant use of strong CYP3A4 enzyme inducers
- Inadequate birth control, pregnancy and/or breast-feeding
- Depression and suicidal ideation
- Patients with severe renal impairment
- Patients with active tuberculosis, serious infections or cancer
- Unexplained and clinically significant weight loss in underweight patients
- Hypersensitivity to the active substance(s) or to any of the excipients
- Hereditary problems of galactose intolerance, lactase deficiency or glucose-galactose malabsorption
- Participating in another trial that might affect the current study or there should be minimum 90 days between participation in another intervention trial

Sample Size: 42 patients

Efficacy Assessments: GELP score, FSDS, DLQI, GHQ-28, PGA, PtGA

Safety Assessments:

- Type, frequency and severity of adverse events. Relationship of adverse events to study drug treatment
- Number of subjects who discontinue study medication due to adverse events
- Increased severity/new manifestations of GELP disease during study
- Weight
- Pregnancy test
- GHQ-28

TABLE OF CONTENTS

CONTACT DETAILS 1
SIGNATURE PAGE 2
PROTOCOL SYNOPSIS 3
TABLE OF CONTENTS 6
LIST OF ABBREVIATIONS AND DEFINITIONS OF TERMS 9
1 INTRODUCTION 10
1.1 Background – Disease 10
1.2 Background - Therapeutic options 10
1.3 Pre-Clinical & Clinical Experience with Investigational Medicinal Product (IMP) 11
1.4 Rationale for the Study and Purpose 12
2 STUDY OBJECTIVES AND RELATED ENDPOINTS 14
2.1 Primary Endpoint 15
2.2 Secondary and Exploratory Endpoints 15
3 OVERALL STUDY DESIGN 15
4 STUDY POPULATION 18
4.1 Selection of Study Population 18
4.2 Inclusion Criteria 18
4.3 Exclusion Criteria 19
5 TREATMENT 19
5.1 Dosage and Drug Administration 20
5.2 Duration of Therapy 20
5.3 Schedule Modifications 20
5.4 Concomitant Medication 21
5.5 Subject Compliance 21
5.6 Drug Accountability 21
5.7 Drug Labeling 21
5.8 Subject Numbering 22
6 STUDY PROCEDURES 23
6.1 Trial flow Chart 23
6.2 By Visit 24
6.3 Withdrawal Visit 26
6.4 After End of Treatment (Follow-up) 26

6.5 Criteria for Treatment Discontinuation 26
6.6 Procedures for Discontinuation 26
6.6.1 Patient Discontinuation 26
6.6.2 Trial Discontinuation 27
6.7 Laboratory Tests 27
7 ASSESSMENTS 27
7.1 Assessment of Efficacy / Response 27
7.2 Safety and Tolerability Assessments 28
7.3 Other Assessments 29
8 SAFETY MONITORING AND REPORTING 30
8.1 Definitions 30
8.1.1 Adverse Event (AE) 30
8.1.2 Serious Adverse Event (SAE) 31
8.1.3 Suspected Unexpected Serious Adverse Reaction (SUSAR) 32
8.2 Expected Adverse Events 32
8.3 Abnormal Laboratory Values 33
8.4 Pregnancy 33
8.5 Recording of Adverse Events 34
8.6 Reporting Procedure 35
8.6.1 AEs and SAEs 35
8.6.2 SUSARs 35
8.6.3 Annual Safety Report 36
8.6.4 Clinical Study Report 36
8.7 Procedures in Case of Emergency 36
9 DATA MANAGEMENT AND MONITORING 36
9.1 Case Report Forms 36
9.2 Source Data 37
9.3 Study Monitoring 37
9.4 Confidentiality 38
9.5 Database management 38
10 STATISTICAL METHODS AND DATA ANALYSIS 39
10.1 Determination of Sample Size 39
10.2 Randomization 39
10.2.1 Allocation- sequence generation 39
10.2.2 Allocation- procedure to randomize a patient 39
10.2.1 Blinding and emergency unblinding 40

slett

slett

10.3 Population for Analysis 40
10.4 Planned analyses 40
10.5 Statistical Analysis 40
10.5.1 Primary analysis 41
10.5.2 Secondary analyses 41
10.5.3 Descriptive statistics 41
10.5.4 Missing data 41
11 STUDY MANAGEMENT 42
11.1 Investigator Delegation Procedure 42
11.2 Protocol Adherence 42
11.3 Study Amendments 42
11.4 Audit and Inspections 42
12 ETHICAL AND REGULATORY REQUIREMENTS 42
12.1 Ethics Committee Approval 42
12.2 Other Regulatory Approvals 42
12.3 Informed Consent Procedure 43
12.4 Subject Identification 43
13 TRIAL SPONSORSHIP AND FINANCING 43
14 TRIAL INSURANCE 44
15 PUBLICATION POLICY 44
16 LIST OF APPENDICES 44
APPENDIX A -BLINDED KIT TEMPLATE 45
17 REFERENCES 48

LIST OF ABBREVIATIONS AND DEFINITIONS OF TERMS

Abbreviation or special term	Explanation
AE	Adverse Event
ALH	Anne Lise Helgesen (Principal Investigator)
CRF	Case Report Form (electronic)
CSA	Clinical Study Agreement
CTU, OUH	Clinical Trial Unit at Oslo University Hospital
DAE	Discontinuation due to Adverse Event
DLQI	Dermatology Life Quality Index
EC	Ethics Committee, synonymous to Institutional Review Board (IRB) and Independent Ethics Committee (IEC)
GCP	Good Clinical Practice
GELP	Genital Erosive Lichen Planus
GHQ-28	General Health Questionnaire
GP	General Practitioner
FSDS	Female Sexual Distress Scale
HCG	Human chorionic gonadotropin
IB	Investigator’s Brochure
ICF	Informed Consent Form
ICH	International Council for Harmonization
IGRA	Interferon-Gamma Release Assays
IMP	Investigational Medicinal Product (includes active comparator and placebo)
KHS	Kristin Helene Skullerud (Investigator)
LP	Lichen planus
OUH	Oslo University Hospital (Oslo Universitetssykehus)
PGA	Physician Global Assessment
PI	Principal investigator
PtGA	Patient Global Assessment
QoL	Quality of Life
REK	Regional Committees for Medical and Health Research Ethics
SAE	Serious Adverse Event
SAP	Statistical Analysis Plan
SLV	Statens legemiddelverk (Norwegian Medicines Agency)
SUSAR	Suspected Unexpected Serious Adverse Reaction
SmPC	Summary of Product Characteristics
SOP	Standard Operating Procedure
UiO	University of Oslo
VAS	Visual Analog Scale
WOCBP	Women of childbearing potential

1 INTRODUCTION

1.1 Background – Disease

Genital erosive lichen planus

Lichen planus (LP) is a chronic autoimmune disease of unknown etiology, affecting skin and mucosal surfaces such as the mouth, esophagus, vulva and vagina. Genital erosive lichen planus is the most common genital subtype.

GELP usually starts in the fifth or sixth decade of life (1) with a slight predominance in women. The disease has a significantly higher impact in females, who need life-long treatment and follow-up. The prevalence of GELP is unknown. Since 2003, app.150 - 200 patients with GELP have been registered in our database at the Vulva Clinic, Oslo University Hospital (OUH).

In women, GELP is characterized by painful vulvo-vaginal erosions and scarring. Scarring may lead to major anatomical changes like absorption of labia and stenosis of the introitus. (2) Total obliteration of the vagina may occur. Normal sexual activity is often impossible.

In our previous case-series study, only 5 out of 58 women with GELP were able to have painless sexual activity.(3) Two-thirds of these patients reported sexual abstinence for several years, having symptoms such as vulvovaginal soreness, irritation, pruritus, bleeding and dyspareunia. The disease consequently has a significant impact on daily living and quality of life. Squamous cell carcinoma may develop within GELP lesions with a prevalence of 3%.(2)

The diagnosis of GELP is based on clinical presentation and/or characteristic histologic findings.(4) Although the cause of the disease is unknown, autoimmune mechanisms seem to be important. Circulating anti-basement zone antibodies have been found in approximately 60 % of patients.(5)

1.2 Background - Therapeutic options

There are few treatment options and no curative treatment for GELP. The number of scientific publications on GELP treatment is low with very few clinical trials.

First-line treatment is topical application of high-potent corticosteroids, such as clobetasol propionate 0,05%, which usually needs to be used life-long.(6) Some patients benefit from the addition of topical tacrolimus. Second-line treatments include systemic immunosuppressant or immune-modifying agents, such as prednisolone, retinoids, methotrexate, cyclophosphamide, azathioprine and cyclosporine.(2, 7) Biologics have been used in a limited number of open treatment studies with inconclusive results.(8) The effect of systemic treatment is often unsatisfactory, and the choice of treatment in GELP is based on empirical evidence and clinical experience only.

A Cochrane Database systematic review from 2013 on interventions for erosive LP affecting mucosal sites found no evidence for the effectiveness of any treatment for GELP and concluded that more RCTs are needed.(9) Recently, our group conducted the first RCT on women with GELP, comparing one session of vulvovaginal photodynamic therapy (PDT) with daily application of high-potent corticosteroids over six weeks.(10) The results were encouraging, giving long-lasting pain reduction after PDT and reduced need of topical corticosteroids. Vulvovaginal PDT requires specialized equipment in gynecological departments.

New and more easily available treatment options are required. An oral systemic treatment will be more accessible and useful for women with moderate or severe GELP.

1.3 Pre-Clinical & Clinical Experience with Investigational Medicinal Product (IMP)

Apremilast is a small-molecule inhibitor of phosphodiesterase 4 (PDE4). Inhibition of PDE4 results in increased intracellular cyclic AMP, which has effect on several inflammatory pathways, resulting in a decrease in pro-inflammatory cytokines, such as TNF-α, interferon-γ, interleukin-23, and an increase in anti-inflammatory cytokines.(11-14)

Apremilast has shown to reduce the severity of inflammatory skin diseases, such as psoriasis and atopic dermatitis.(15, 16) Currently, the effect of apremilast on other autoimmune skin diseases is being investigated in several studies.(17) Apremilast has also been found to be effective in treating oral ulcers in a placebo-controlled study on Behçet's disease, a disease with oral and genital ulcers as key features.(12) A pilot study on apremilast in cutaneous LP concludes that apremilast may be efficacious in the treatment of LP, but double-blind, controlled trials are necessary to thoroughly evaluate its safety and efficacy.(18) More recently, case reports showing effect of apremilast on oral LP and LP-associated stenotic esophagitis have been published.(19-21)

With increasing evidence of effect in other cutaneous and mucosal inflammatory diseases, apremilast may represent a potential new treatment for GELP. One important advantage of apremilast is its favorable safety profile and generally tolerable side effects, which is essential for chronic diseases requiring continuous treatment. Common adverse effects include short-term nausea, diarrhea, headache, and, less commonly, moderate weight loss and upper respiratory tract infections.(22, 23) In a placebo-controlled trial in patients with psoriasis and/or psoriatic arthritis, depression or depressed mood was reported by 1.0-1.3% among apremilast-treated patients versus 0.4-0.8% among placebo-treated patients.(24) The risks and benefits of apremilast treatment should be evaluated carefully prior to treatment in patients with a history of depression and/or suicidal thoughts or behavior, and close monitoring for worsening of such events during therapy is advised. (24, 25)

Worldwide, more than 160,000 patients have been treated with apremilast (December 2016). (26) It is approved for use in several regions throughout the world and accepted for reimbursement in psoriasis in most EU countries. In Norway, apremilast is currently approved for use in chronic plaque psoriasis and psoriatic arthritis

1.4 Rationale for the Study and Purpose

Genital erosive lichen planus (GELP) is a chronic, inflammatory and scarring genital disease. The disease may have a significant impact on daily living, quality of life and sexual function. There is a considerable lack of high-quality evidence on treatment options for GELP and few effective therapeutic facilities are available in current clinical practice.(2)

The aims of our study are to investigate the clinical and immunohistochemical effects of a new oral anti-inflammatory treatment, apremilast, for women with moderate-to-severe GELP in a double-blinded, randomized, placebo-controlled trial (RCT). Apremilast is an inhibitor of phosphodiesterase 4 (PDE4) with documented effect in several inflammatory skin diseases, but it has not yet been studied in patients with GELP. We will also investigate quality of life and sexual function in GELP patients by using validated questionnaires, as no larger quality-of-life studies have been performed in similar patient groups. The study design was chosen based on relevant experience from other studies on apremilast.

The drug dose is equivalent to the dose used for approved indications. We have no evidence supporting a different dose of apremilast being more effective for GELP. If apremilast becomes a treatment option for genital lichen planus in clinical practice at a later stage, 30 mg will be the dose available for prescription.

The choice of study design is supported by the lack of effective treatment options in GELP and need for more randomized controlled trials (RCTs) described in literature and in the Cochrane report cited above.

In GELP, topical super-potent steroid ointment (clobetasol propionate or similar) is the first line treatment. As a substantial number of patients will not have satisfying symptom relief, and complications by this treatment, there is a need for additional treatment options. Systemic treatments may be beneficial especially in women with multiple site-disease. Currently, there is no evidence-based guidance on the use of systemic treatment in GELP.

Placebo has been chosen as the comparator treatment to control for as many potential influences of the effect of the interventional drug as possible.

Although the patients in this study are not prescribed clobetasol propionate according to a specific treatment regime, all participants are allowed to use this treatment as needed according to symptoms and will record the frequency of use weekly.

Based on this, we have chosen to test apremilast against placebo, as no proven effective systemic treatment exists, and the interventional treatment (apremilast) and placebo are added on to common topical therapy. In the add-on study design, subjects in the placebo group will not be subject to additional risks of serious or irreversible harm as a result of not receiving the best proven intervention.(27)

Although large placebo-controlled studies on apremilast showed efficacy at primary endpoint at week 16(23, 28), the SmPC states that the greatest improvement in pivotal trials was observed within 24 weeks. As a consequence, treatment in clinical practice should be reconsidered if there is no therapeutic benefit after 24 weeks. Based on this recommendation and in order to maximize the potential of demonstrating therapeutic response, we have chosen the primary end point at 24 weeks.

Risks/ benefits

Different benefits and risks are associated with the proposed study regime of apremilast in psoriasis and psoriatic arthritis. Known effects of apremilast are summarized in 1.3. Potential benefits in GELP are reduced pain and inflammation, reduced sexual distress and improved quality of life.

According to the SmPC, the most commonly reported adverse reactions in Phase III clinical studies have been gastrointestinal (GI) disorders including diarrhea (15.7%) and nausea (13.9%). These GI adverse reactions were mostly mild to moderate in severity, with 0.3% of diarrhea and 0.3% of nausea reported as being severe. These adverse reactions generally occurred within the first 2 weeks of treatment and usually resolved within 4 weeks. The other most commonly reported adverse reactions included upper respiratory tract infections (8.4%), headache (7.9%), and tension headache (7.2%). Overall, most adverse reactions were considered to be mild or moderate in severity. Patients 65 years of age or older may be at a higher risk of complications.

Of special concern for GELP patients is diarrhea, which may cause additional irritation/ inflammation of the genital area during the first weeks of treatment. Patients will be given advice on hygiene measures and use of topical treatments such as moisturizing and barrier creams at initiation of treatment to reduce this.

Psychiatric adverse effects have been reported with apremilast treatment. The SmPC describes symptoms of depression as common (≥1/100 to <1/10). Post-marketing studies and clinical studies have reported uncommon cases of suicidal ideation and behaviour. The exclusion criteria reflects this, and measures to prevent psychiatric side effects are listed in table 1 and described further in Ch. 8.

Table 1. Risk/benefit monitoring and measures

**Risk factors:**	**Monitoring/measures:**
**Diarea, nausea, vomiting**	Information about prophylactic procedures and treatment at study start<br>First follow-up after 4 weeks<br>Weight measures every follow-up
**Infections**	Information sent to the GP at study start
**Psychiatric symptoms**	GHQ 28 at every visit<br>Phone calls/visits every 4 weeks<br>Contact study personnel by phone or e-mail 24 hours<br>Known depression is an exclusion criterium<br>Information sent to the GP at study start<br>Avoid concomitant medication that increase risk
**Benefits:**	**Monitoring/measures:**
Reduced pain	GELP score, PGA, PtGA
Reduced inflammation	GELP score, photos
Reduced sexual distress	DFDS score, PtGA
Improved quality of life	QOL score, PtGA

2 STUDY OBJECTIVES AND RELATED ENDPOINTS

The main objective of this trial is to assess the efficacy of apremilast in the treatment of GELP in women.

Table 2 describes the relation between objectives and endpoint.

Table 2

	Objectives	Endpoints
**Primary**	To assess the effect of apremilast treatment in women with GELP	**Primary**<br>• Mean GELP score at week 24 in apremilast-treated patients versus placebo-treated patients<br>**Secondary**<br>• Mean GELP score improvement from week 0 to week 24 in all patients<br>• Weekly use of topical steroid, collected from patient diary<br>• Weekly VAS pain score, collected from patient diary<br>• Number of patients with GELP score improvement at week 16 and 24<br>• Separate GELP score assessments: Area of involvement (in cm^2), number of erosions, erythema, striae and pain (VAS) at week 4, 16 and 24<br>• Physician Global Assessment (PGA) and Patient Global Assessment (PtGA) scores at week 0, 16 and 24<br>**Exploratory**<br>• Description of extragenital lichen planus at week 0, 16 and 24<br>• Clinical photos taken at week 0 and week 24
**Secondary**	To assess quality of life in women with GELP before and during apremilast treatment	Selected QoL measures (DLQI, GHQ-28) at week 0, 16 and 24
	To assess sexual function in women with GELP before and during apremilast treatment	Sexual function assessments (FSDS-R) at week 0, 16 and 24
Exploratory	To describe immune histochemical changes and expression of selected cytokines in GELP lesions after treatment with apremilast	• Description of immune histochemical changes and expression of selected cytokines before and after apremilast therapy, assessed in vulvar or vaginal biopsies at week 0 and 24

2.1 Primary Endpoint

1. Mean GELP score at week 24 in apremilast-treated patients versus placebo-treated patients

2.2 Secondary Endpoints

2. Mean GELP score improvement from week 0 to week 24 in all patients
3. Weekly use of topical steroid, collected from patient diary
4. Weekly VAS pain score, collected from patient diary
5. Number of patients with GELP score improvement at week 16 and 24
6. Separate GELP score assessments: Area of involvement (in cm^2), number of erosions, erythema, striae and pain (VAS) at week 4, 16 and 24
7. Physician Global Assessment (PGA) and Patient Global Assessment (PtGA) scores at week 0, 16 and 24
8. Selected QoL measures (DLQI, GHQ-28) at week 0, 16 and 24
9. Sexual function assessments (FSDS-R) at week 0, 16 and 24

Exploratory endpoints

10. Description of immune histochemical changes and expression of selected cytokines before and after apremilast therapy, assessed in vulvar or vaginal biopsies at week 0 and 24
11. Description of extragenital lichen planus at week 0, 16 and 24
12. Clinical photos taken at week 0 and week 24
13. Adverse events

3 OVERALL STUDY DESIGN

There are very few controlled studies on treatment of GELP in women. The Cochrane Database systematic review on treatment of erosive lichen planus affecting mucosal sites(9) concludes that more RCTs on a larger scale are needed in the oral and genital ELP populations and suggest that future studies should have standardized outcome variables that are clinically important to affected individuals.

This is a phase 2, single-center, randomized, placebo-controlled, double-blind, parallel-group study.

An equal number of participants are randomized to apremilast- or placebo treatment arms. Based on experience from previous studies on apremilast used for other indications, effect is usually achieved within 3-4 months,(29) with some ecpected additional effect for another two months.

Screening will be performed no more than 90 days prior to baseline visit. Patients will receive information on participation at screening visit.

Patients who qualify for participation according to inclusion criteria and who have signed the informed consent form will be included at baseline visit (day 0). They will be randomized at inclusion to receive either study medication (apremilast) or placebo. Screening and baseline visit may be on the same day, but preferably patients will have at least 24 hours between screening and baseline to allow adequate time for patient information/ informed consent.

Randomization will be performed in the electronic data capture system Viedoc which includes a randomization module and both patients and clinicians will be blinded for the allocation. The study is double-blinded for the randomization throughout the treatment period and until end of trial.

Following randomization, 21 patients will receive study medication (oral apremilast) and 21 patients will receive oral placebo. Initial standard titration of dose day 1-6 is followed by standard dose of 30 mg apremilast b.i.d. or placebo b.i.d for a total period of 24 weeks. Tablet blister cards are identical in appearance in both groups.

**Study Period**	First patient enrolled: 24.09.2019<br>Anticipated recruitment period: 01.03.2019 – 01.07.2021<br>Estimated date of last patient completed: 31.12.2021
**Treatment Duration:**	24 weeks.<br>The end of the trial is defined as the date of the last vist of the last patient undergoing the trial.
**Follow-up:**	At the end of study at week 24 or if earlier withdrawal from study, patients will be given an appointment with one of the participating clinicians (ALH or KHS) for regular clinical follow-up after approximately 3-6 months.

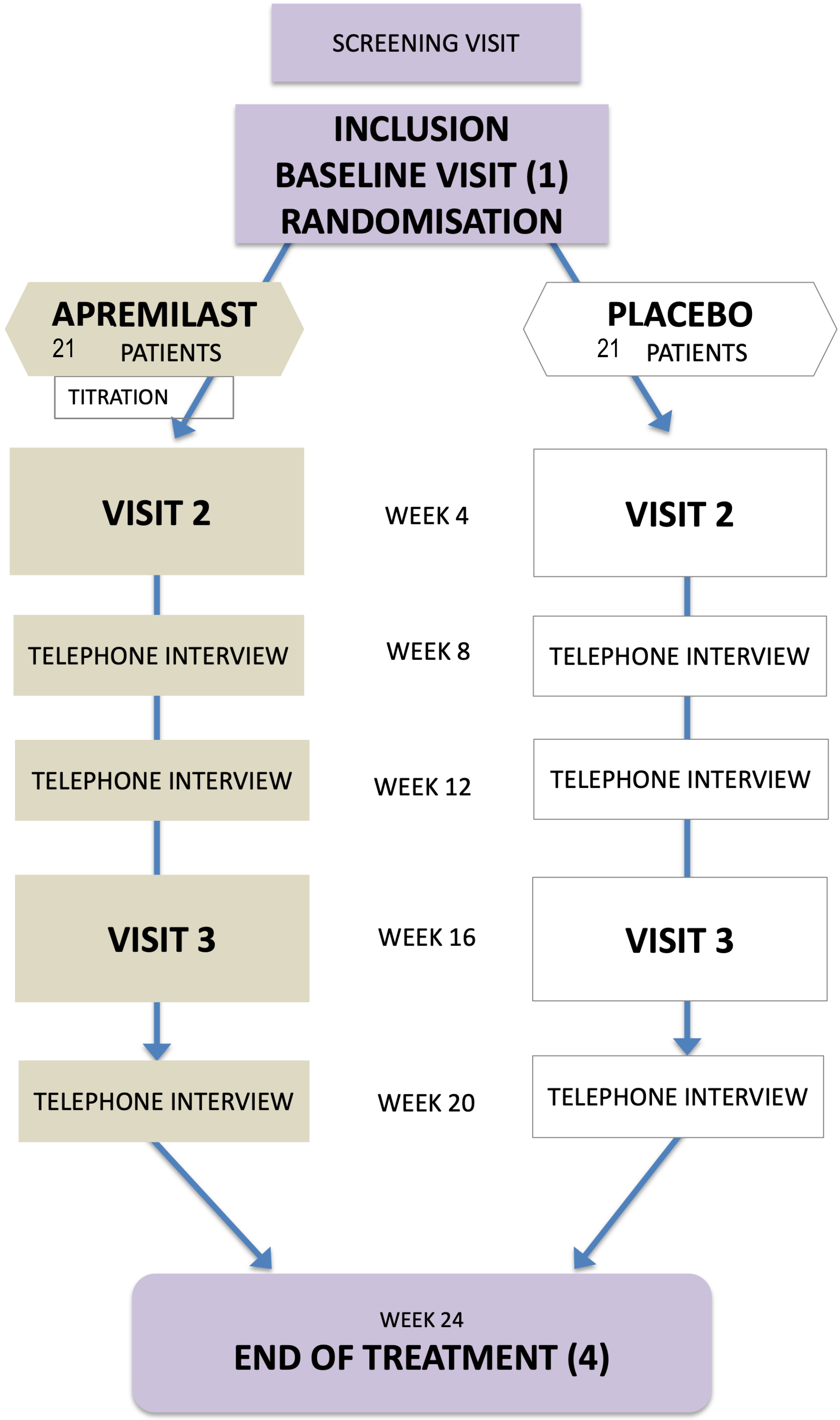
SCREENING VISIT
INCLUSION
BASELINE VISIT (1)
RANDOMISATION
APREMILAST
21 PATIENTS
TITRATION
PLACEBO
21 PATIENTS
VISIT 2
WEEK 4
VISIT 2
TELEPHONE INTERVIEW
WEEK 8
TELEPHONE INTERVIEW
TELEPHONE INTERVIEW
WEEK 12
TELEPHONE INTERVIEW
VISIT 3
WEEK 16
VISIT 3
TELEPHONE INTERVIEW
WEEK 20
TELEPHONE INTERVIEW
WEEK 24
END OF TREATMENT (4)

Figure 1.

4 STUDY POPULATION

4.1 Selection of Study Population

The Vulva Clinic at Oslo University Hospital, Oslo, Norway is a national tertiary referral center for women with chronic vulval diseases. In 2015, more than 700 consultations were carried out, with approximately half of the patients living in the southeast region of Norway.

150-200 patients with genital lichen planus are registered in the clinic database. 4 specialists, 2 gynecologists and 2 dermatologists (ALH and KHS) are employed and collaborate with other health care professionals. Study participants will be recruited mainly from the Vulva Clinic but may also be referred from other specialized vulva services and specialists outside OUH.

Number of Patients
42 patients will be included in this trial, 21 subjects in active treatment group and 21 subjects in placebo group.

4.2 Inclusion Criteria

All of the following conditions must apply to the prospective patient at screening prior to receiving study medication:

- >18 years of age
- Female
- Moderate to severe GELP with the diagnosis based on characteristic clinical and/or histological features. Minimum GELP score 5/30 in vagina and/or vulva (scored separately), of which erythema and pain ≥1 are mandatory
- Sexually active patients of childbearing potential must agree to use highly effective contraception during treatment and 28 days after the last dose of IMP
- Signed informed consent and expected cooperation of the patients for the treatment and follow up is obtained and documented according to ICH GCP, and national/local regulations.

  A woman is considered of childbearing potential (WOCBP), i.e. fertile, following menarche and until becoming post-menopausal unless permanently sterile. Permanent sterilization methods include hysterectomy, bilateral salpingectomy and bilateral oophorectomy. A postmenopausal state is defined as no menses for 12 months without an alternative medical cause.

  Highly effective birth control methods: (36)

  - combined (estrogen and progestogen containing) hormonal contraception associated with inhibition of ovulation; oral, intravaginal or transdermal
  - progestogen-only hormonal contraception associated with inhibition of ovulation, oral, injectable or implantable
  - intrauterine device
  - intrauterine hormone-releasing system
  - bilateral tubal occlusion

- vasectomized partner, provided that partner is the sole sexual partner of the WOCBP trial participant and that the vasectomized partner has received medical assessment of the surgical success
- sexual abstinence, defined as refraining from heterosexual intercourse during the entire period of risk associated with the study treatments.

4.3 Exclusion Criteria

Patients will be excluded from the study if they meet any of the following criteria:

- Patients receiving other systemic immune modulating therapy. Immune modulating therapy other than the study drug is prohibited during the study. Subjects who have been taking the following medications must adhere to the following minimum washout periods:

  - Four weeks prior to the day of inclusion for non-biologic systemic immunosuppressive agents
  - Twelve weeks prior to the day of inclusion for adalimumab, infliximab, etanercept and similar agents
  - Twelve months prior to the day of inclusion for rituximab.

- Concomitant use of strong CYP3A4 enzyme inducers. Apremilast exposure is decreased when administered concomitantly with strong inducers of CYP3A4 and may result in reduced clinical response. The use of strong CYP3A4 enzyme inducers (e.g. rifampicin, phenobarbital, carbamazepine, phenytoin and St. John's Wort) with apremilast is not recommended.(29)
- Inadequate birth control, pregnancy and/or breast-feeding
- Depression and suicidal ideation (GHQ-28 score more than 7/28)
- Patients with severe renal impairment (creatinine clearance of less than 30mL per minute) as described in SmPC
- Patients with active tuberculosis, serious infections or cancer
- Unexplained and clinically significant weight loss in underweight patients
- Hypersensitivity to the active substance or to any of the excipients (listed in SmPC)
- Hereditary problems of galactose intolerance, lactase deficiency or glucose-galactose malabsorption
- Participating in another trial that might affect the current study or there should be minimum 90 days between participation in another intervention trial

5 TREATMENT

For this study apremilast and placebo are defined as Investigational Medicinal Products (IMP). Apremilast is approved for use in Norway but for another indication (psoriasis and psoriatic arthritis). Subjects will receive a standard dose of 30 mg BID.

Placebo and active study medication will be manufactured and supplied to the investigators by Celgene Corporation. Import will be performed by The Hospital Pharmacy.
Apremilast will be supplied as 10, 20, or 30 mg tablets for oral administration. Additional description of the study drug can be found in the Investigator's Brochure.

After import and registration by the pharmacy, the IMPs will be stored in the pharmacy in a locked, safe area with temperature data logging and access limited to designated study personnel or pharmacists. Apremilast should not be stored above 30ºC.

The IMP will be packaged in blister cards and labelled by Celgene and will be administered to the subjects by authorized pharmacy personnel.

There will be sufficient medication in the blister cards to cover up to 1-month delay in the scheduled assessments.

Note: Following Celgene's sale to Amgen of the global rights to Otezla®, rights and obligations under the contract with Celgene is transferred to Amgen fra May 1, 2020.

5.1 Dosage and Drug Administration

Apremilast 30 mg and placebo tablets are administered orally, twice daily (morning and evening), approximately 12 hours apart. To reduce risk of gastrointestinal symptoms, dose titration day 1-6 is recommended and apremilast will be administered according to the following schedule:

Day 1: 10 mg in morning

Day 2: 10 mg in morning and 10 mg in evening

Day 3: 10 mg in morning and 20 mg in evening

Day 4: 20 mg in morning and 20 mg in evening

Day 5: 20 mg in morning and 30 mg in evening

Day 6 and thereafter: 30 mg twice daily

This is similar to the standard dosage regimen for psoriasis and psoriatic arthritis.

For details on dosing regime and tablet kits refer to appendix 1, blinded kit template.

5.2 Duration of Therapy

The IMP will be given for 24 weeks.

5.3 Schedule Modifications

Participants will not be able to reduce apremilast (or placebo) dose in the study.

If a subject misses 4 or more consecutive days of dosing, the PI should be contacted for further instruction. The investigator will decide on further treatment with the IMP based on clinical

evaluation, and the dose will be registered in the CRF. Criteria for treatment discontinuation are listed in 6.5.

5.4 Concomitant Medication

5.4.1. All systemic medication (incl. vitamins, herbal preparation and other "over-the-counter" drugs) used by the patient will be recorded in the patient's file and CRF. Prohibited concomitant medications are listed in the exclusion criteria.
Concomitant treatment with other medicinal products that are likely to increase the risk of psychiatric events should be evaluated carefully and preferably be avoided.

Topical medications: In the genital area**,** patients are allowed to use potent topical steroid (clobetasol propionate or similar) as needed during the study. They will record frequency of use per week in the patient's diary. Study subjects are not allowed to use other immunomodulating topical medications in the genital area.

5.5 Subject Compliance

Study subjects will record any interruptions of dosage in the weekly patient's diary and will be asked about compliance at each study visit/ telephone consultation.

5.6 Drug Accountability

The responsible site personnel will use the study drug only within the framework of this clinical study and in accordance with this protocol. The hospital pharmacy is responsible for import and confirmation of receipt of study drugs, documentation of batches received and that the study drugs are securely stored under the advised temperature conditions. Study subjects will receive information on which tablet blister card no. they have been assigned to by the Viedoc system, and in what order the blister cards should be used, by the study team at visits. They will then collect the corresponding blister cards at the hospital pharmacy. The pharmacist dispensing the drugs will be responsible for the registration of batches delivered to patients. When a patient returns for the next visit, she will bring back the blister cards and any unused medication will be registered by the study team in the e-CRF and collected by the study team who will then return it to the pharmacy. At the return of study medication to the hospital pharmacy, the pharmacy will be responsible for destruction or disposal of any unused medicinal product and for recording this.

5.7 Drug Labeling

The investigational product will have a label permanently affixed to the outside and will be labeled in Norwegian, in accordance with ICH GCP and national regulations. Labels will be prepared and affixed by Celgene after approval by KHS/ ALH.

Label will include:
a) Sponsor name, address and telephone number
b) Name and telephone number of Principal Investigator (main contact)
c) Information on pharmaceutical dosage form/ dosing instructions
d) Batch number
   Protocol number Celgene: AP-CL-LICPLAN-PI-12824
   Protocol number sponsor: APGELP
   EudraCT No: 2017-004116-19
e) Patient's enrolment code/ subject number
f) Patient's initials

g) Name of prescribing doctor
h) Date dispensed
i) Period of use (expiry date)
j) Storage conditions
k) “For clinical trial use only”
l) “Keep out of reach of children”
m) Kit number
e)-h) will be blank spaces filled in by the investigators or pharmacist before dispensing the IMP to study subjects.

Note: Following Celgene’s sale to Amgen of the global rights to Otezla®, rights and obligations under the contract with Celgene is transferred to Amgen fra May 1, 2020.

5.8 Subject Numbering

A unique subject number that is assigned when the subject signs the Informed Consent Form identifies each subject in the study. Once assigned, the subject number cannot be reused for any other subject.

6 STUDY PROCEDURES

6.1 Table 3. Trial flow Chart

Visits/ procedure	Screening	Baseline Inclusjon	Visit 2	Telephone interview	Telephone interview	Visit 3	Telephone interview	End of treatment visit	Withdrawal visit
**Week +/- 7 days**	Max -90 days	0	4	8	12	16	20	24	Max 14 days after
**Informed consent**	x								
**Eligibility assessment**	x								
**Randomisation**		x							
**Compliance**			x	x	x	x	x	x	x
**Medical history**	x	x	x			x		x	x
**Safety evaluation**	x	x	x	x	x	x	x	x	x
**Clinical exmination**	x	x	x			x		x	x
**Weight**	x	x	x			x		x	x
**PGA**		x				x		x	x
**PtGA**		x				x		x	x
**DLQI**		x				x		x	x
**FSDS-R**		x				x		x	x
**GHQ-28**	x	x	x			x		x	x
**GELP score**	x	x	x			x		x	x
**Vulval/ vaginal biopsy**		x						x	
**Hcg**[1]	x	x	x	x	x	x	x	x	x
**GFR**[1]	x								
**IGRA**[1]	x								
**Collect patient diary**						x		x	
**Clinical photo**		x						x	

[1] Blood or urine (HCG) sample

6.2 By Visit

The study comprises the following visits, study procedures and assessments:

Screening:
Medical history, eligibility assessment
Clinical examination
Weight
Safety evaluation
GELP, GHQ-28. HCG, GFR, IGRA
Informed consent

Baseline visit (week 0):
Randomization and initiating apremilast or placebo treatment
Medical history including current use of topical steroid (no of days used per week last 4 weeks).
Clinical examination
Weight
Safety evaluation
Clinical photo
PGA, GELP, PtGA, FSDS-R, DLQI, GHQ-28
Vulval and/ or vaginal biopsy

Visit 2 (week 4):
Compliance, medical history
Clinical examination
Safety evaluation
Weight
GELP, GHQ-28

Telephone interview (week 8): Compliance, safety evaluation

Telephone interview (week 12): Compliance, safety evaluation.

Visit 3 (week 16):
Compliance, medical history.
Clinical examination
Weight
Safety evaluation
PGA, GELP, DLQI, FSDS-R, GHQ-28, PtGA
Collect patient diary

Telephone interview (week 20): Compliance, safety evaluation

Visit 4 (week 24):
Compliance, medical history
Clinical examination
Weight
Safety evaluation
Clinical photo

PGA, GELP, PtGA, DLQI, FSDS-R, GHQ-28
Collect patient diary
Vulval/vaginal biopsy

Informed consent
Each subject must have given informed consent voluntarily before any study specific procedures are initiated.

Study concept/method
Inclusion will take place in a 12 months period. Patients must sign consent forms to participate and will receive oral and written information about the project and their right to withdraw from the study at any time.

Before enrolment patients will be screened for eligibility. Case history and use of medication will be registered. During all visits, clinical examination and safety monitoring will be performed. Extragenital LP will be registered.

Treatment phase
After inclusion, patients will be randomized to either oral apremilast or matching placebo, starting at week 0. After initial standard titration of dose day 1-6, standard dose of 30 mg apremilast b.i.d. or placebo is used for 24 weeks. Patients and investigators are blinded to treatment allocation throughout the study period.

Telephone interviews:
At week 8, 12 and 20, participants will be contacted by phone by a research nurse and asked about possible side effects, compliance, and will be encouraged to fill in the diary log. The interview will specifically focus on any psychiatric side effects. For WOCBP, the result of the home pregnancy test will be recorded.

Clinical assessments
Clinical assessments will consist of registering GELP score, clinical photos of target area, and Physician Global Assessment (PGA) score(30) for evaluation of the clinical grade of GELP. In addition, Patient's Global Assessment (PtGA)(30), Dermatology Life Quality Index (DLQI) score, General Health Questionnaire (GHQ-28) and Female Sexual Distress Scale (FSDS-R) will be recorded.

Clinical status/ examination:
- Inspection of the genital area, the cavum oris and, if relevant, other affected skin areas
- Blood pressure
- Cardiac and pulmonary auscultation

Patients' weekly assessment: All patients will receive a diary at baseline for weekly registration of steroid use (no. of days per week), genital pain assessment (VAS scale 0-10) and adverse events. The diary is collected at week 16 and week 24. Partcipants may also use ViedocMe weekly for electronic registration.

6.3 Withdrawal Visit

All patients who withdraw from the study will be offered an appointment with one of the participating clinicians (ALH or KHS) 7-14 days after withdrawal. If the patient is willing to state her reason for withdrawal, the reason for withdrawal will be registered.

6.4 After End of Treatment (Follow-up)

At the end of study at week 24 or if earlier withdrawal from study, patients will be given an appointment with one of the participating clinicians (ALH or KHS) after approximately 3-6 months, for regular clinical follow-up.

6.5 Criteria for Treatment Discontinuation

Patients may be discontinued from study treatment and assessments at any time. Specific reasons for discontinuing a patient for this study are:

- Voluntary discontinuation by the patient who is at any time free to discontinue his/her participation in the study, without prejudice to further treatment
- Patient lost to follow-up
- Reasons as judged by the Principal Investigator to compromise the safety and well-being of the patient by continued participation in the trial

Reasons for discontinuation of study treatment, but not assessments, are:

- Safety reasons as judged by the Principal Investigator
- Incorrect enrolment, i.e. the patient does not meet the required inclusion/exclusion criteria for the study
- A patient becoming pregnant

Procedures for Discontinuation

6.5.1 Patient Discontinuation

Patients who withdraw or are withdrawn from the study, will stop further treatment with the IMP. The reason for discontinuation shall be recorded if the patient agrees. Patients who are withdrawn from the study will be offered a final assessment (end of study visit) and will continue standard clinical follow-up at the Vulva clinic.

The investigators will follow up any significant adverse events until the outcome either is recovered or resolved, recovering/resolving, not recovered/not resolved, recovered/resolved with sequelae, fatal or unknown.

All patients randomized will be included in the study population. Patients who withdraw or are withdrawn from the study after randomization cannot be replaced.

Patients who are withdrawn from treatment will continue study visits and assessments as described in this protocol.

6.5.2 Trial Discontinuation

The whole trial may be discontinued at the discretion of the PI or the Sponsor in the event of any of the following:

- Occurrence of AEs unknown to date in respect of their nature, severity and duration
- Medical or ethical reasons affecting the continued performance of the trial
- Difficulties in the recruitment of patients
- Cancellation of drug development

The Sponsor and Principal Investigator(s) will inform all investigators, the relevant Competent Authorities and Ethics Committees of the termination of the trial along with the reasons for such action. If the study is terminated early on grounds of safety, the Competent Authorities and Ethics Committees will be informed within 15 days.

6.6 Laboratory Tests

Collection of blood samples will be performed in accordance with OUH laboratory standard procedures.

7 ASSESSMENTS

7.1 Assessment of Efficacy / Response

GELP score:
The GELP score has been developed as a clinical composite grading tool for use in the recent study on vulvovaginal PDT in GELP by one of the investigators (ALH).(10) The GELP score includes a severity assessment in four out of five clinical diagnostic criteria for GELP included in an international, web-based Delphi consensus report.(4) Area of genital involvement, erythema, striae, number of erosions and pain are registered and scored 0-3 at all clinical visits.

Table 4. GELP score: Scoring system for clinical assessment of genital erosive lichen planus (GELP) in women. Vulval and vaginal involvement is assessed separately, resulting in a maximum GELP score of 30.

Area of involvement	None<br>< 3 cm<br>3–6 cm<br>> 6 cm	0<br>1<br>2<br>3
Intensity of erythema	None<br>Mild<br>Moderate<br>Strong	0<br>1<br>2<br>3
Number of erosions	None<br>1<br>2–3<br>>3	0<br>1<br>2<br>3
Striae	None<br>Minimal<br>Moderate<br>Extensive	0<br>1<br>2<br>3
Pressure-induced pain<br>(Visual analogue scale 1–10)	None<br>1–3<br>4–6<br>7–10	0<br>1<br>2<br>3

- **VAS:** VAS is recorded on a visual linear scale ranging from 0 (no pain) to 10 (worst imaginable pain)
- **PGA score:** 5-point disease severity scoring system, assessed by the clinician

  0 - Clear

  1 - Almost clear

  2 - Mild

  3 - Moderate

  4 - Severe

- **PtGA:** 5-point disease severity scoring system (as described for PGA), assessed by the patient
- **Topical steroid use:** Frequency of application is recorded in the patient diaries as the number of days being used per week.

7.2 Safety and Tolerability Assessments

Safety will be monitored through the assessments described below as well as registration of AEs at each visit. Significant findings that are present prior to the signing of informed consent will be included in the relevant medical history/ current medical condition page of the CRF. For details on AE collection and reporting, see section 8.

For the assessment schedule, see flow chart in section 6.1, and table 1.

Physical examination will include

- Inspection of the genital area, the cavum oris and, if relevant, other affected skin areas.
- Blood pressure.
- Cardiac and pulmonary auscultation.

Body weight will be measured in indoor clothing, without shoes.

GHQ-28 will serve as a screening device to assist the assessment of negative psychological effects of study medication.

Pregnancy tests (in WOCBP) will be performed monthly, at study visits or by the patient at home. The result will be reported to study personnel monthy.

7.3 Other Assessments

Skin/ mucosal biopsies:
Punch biopsies of 4 mm will be taken from GELP lesions in patients in both treatment arms at week 0 and week 24. Appropriate immunohistochemical methods will be used, and expression of selected pro-inflammatory and anti-inflammatory mediators will be described.

The assessments will be performed in a blinded manner by the the Department of Pathology, Oslo University Hospital.

Patients will be informed about the biopsy procedure in the patient information letter. If any of the study subjects decline this procedure, they will not be excluded from the study.

Quality of life questionnaires:
Three validated QoL questionnaires will be used. To be able to compare the results with studies in other vulvar and/or mucosal inflammatory diseases, we have chosen questionnaires that have been used in previous GELP QoL studies(31, 32):

DLQI – Dermatological Life Quality Index
FSDS – Female Sexual Distress Scale
GHQ28 – General Health Questionnaire

DLQI is a widely used questionnaire aiming to measure the impact of skin disease on adult patients' quality of life. The DLQI consists of 10 items covering six basic topics: symptoms and feelings, daily activities, leisure, work or school, personal relationships, and treatment.(33)

FSDS is a standardized 13-item scale for assessment of sexually related personal distress in women. It may serve as a screening tool to identify women with high and low sexual function.(34)

GHQ28 is a screening device for identifying minor psychiatric disorders. It is a 28-item scaled version assessing somatic symptoms, anxiety and insomnia, social dysfunction and severe depression.(35)

The results will be compared with the results from studies in patients with genital psoriasis, lichen sclerosus and vulvar dermatitis.

8 SAFETY MONITORING AND REPORTING

The Investigator is responsible for the detection and documentation of events meeting the criteria and definition of an adverse event (AE) or serious adverse event (SAE). During the study period, study personnel communicate with patients monthly, either as patient visit or a phone call.

If there is a life-threatening or acute health problem, the patient must seek standard emergency care in the health system.

Participants will receive a patient card with contact information and information on participation in the study and will be able to contact study personnel by phone or e-mail 24 hours a day throughout the treatment period. Study personnel will have online access to the e-CRF. Emergency unblinding will be possible with online access but will be restricted to situations where this is vital for the patient's health or further treatment (ref. 10.2.1). Each patient will be instructed to contact the investigator immediately should they manifest any signs or symptoms they perceive as serious.

At inclusion the patient's General Practitioner (GP) will be sent written information of the study and potential adverse effects. Concerning potential psychiatric side effects of the IMP, patients will be encouraged to inform family/caregiver about this risk and immediately inform their GP or emergency health service of changes in behaviour or mood, and of any suicidal thoughts.

The methods for collection of safety data are described below in 1.4 and table 1.

8.1 Definitions

8.1.1 Adverse Event (AE)

An adverse event (AE) is any noxious, unintended, or untoward medical occurrence that may appear or worsen in a subject during the course of a study. It may be a new intercurrent illness, a worsening concomitant illness, an injury, or any concomitant impairment of the subject's health, including laboratory test values, regardless of etiology. Any worsening (i.e., any clinically significant adverse change in the frequency or intensity of a pre-existing condition) should be considered an AE. A diagnosis or syndrome should be recorded on the AE page of the CRF rather than the individual signs or symptoms of the diagnosis or syndrome.

Abuse, withdrawal and overdose (accidental or intentional) to an investigational product should be reported as an AE. Any sequela of an accidental or intentional overdose of an investigational product should be reported as an AE or serious adverse events (SAE) . In the event of overdose, the subject should be monitored as appropriate and should receive supportive measures as necessary. Actual treatment should depend on the severity of the clinical situation and the judgment and experience of the treating physician.

All subjects will be monitored for AEs during the study. Assessments may include monitoring of any or all of the following parameters: the subject's clinical symptoms, laboratory, pathological, physical examination findings, or findings from other appropriate tests and procedures.

All AEs will be recorded by the Investigator from the time the subject signs informed consent to at least 28 days after the last dose of IMP or until the last study visit, whichever period is longer

as well as those serious adverse events (SAEs) made known to the Investigator at any time thereafter that are suspected of being related to the IMP. SAEs occurring after signing the ICF but prior to treatment will also be recorded.

All AEs and SAEs will be recorded on the AE page of the CRF and in the subject's source documents.

The term AE is used to include both serious and non-serious AEs.

If an abnormal laboratory value/vital sign are associated with clinical signs and symptoms, the sign/symptom should be reported as an AE and the associated laboratory result/vital sign should be considered additional information that must be collected on the relevant CRF.

During the course of the study all AEs and SAEs will be proactively followed up for each patient; events should be followed up to resolution, unless the event is considered by the investigator to be unlikely to resolve due to the underlying disease. Every effort should be made to obtain a resolution for all events, even if the events continue after discontinuation/study completion.

8.1.2 Serious Adverse Event (SAE)

A serious adverse event (SAE) is any AE occurring at any dose that:

- Results in death;
- Is life-threatening (i.e., in the opinion of the Investigator, the subject is at immediate risk of death from the AE);
- Requires inpatient hospitalization or prolongation of existing hospitalization (hospitalization is defined as an inpatient admission, regardless of length of stay);
- Results in persistent or significant disability/incapacity (a substantial disruption of the subject's ability to conduct normal life functions);
- Is a congenital anomaly/birth defect;
- Constitutes an important medical event.

Important medical events are defined as those occurrences that may not be immediately life threatening or result in death, hospitalization, or disability, but may jeopardize the subject or require medical or surgical intervention to prevent one of the other outcomes listed above. Medical and scientific judgment should be exercised in deciding whether such an AE should be considered serious. In such situations, or in doubtful cases, the case should be considered as serious.

Events **not considered** to be SAEs are hospitalizations for:

- Routine treatment or monitoring of the studied indication not associated with any deterioration in condition.
- A procedure for protocol/disease-related investigations. However, hospitalization or prolonged hospitalization for a complication of such procedures remains a reportable SAE.
- Hospitalization or prolongation of hospitalization for technical, practical, or social reasons, in absence of an AE.

- A procedure that is planned (i.e., planned prior to starting of treatment on study); must be documented in the source document and the CRF. Hospitalization or prolonged hospitalization for a complication remains a reportable SAE.
- An elective treatment of a pre-existing condition unrelated to the studied indication.
- Emergency outpatient treatment or observation that does not result in admission, unless fulfilling other seriousness criteria above.

If an AE is considered serious, both the AE page and the SAE Report Form of the CRF must be completed.

For each AE/SAE, the Investigator will provide information on severity, start and stop dates, relationship to IMP, action taken regarding IMP, and outcome.

8.1.3 Suspected Unexpected Serious Adverse Reaction (SUSAR)

Suspected Unexpected Serious Adverse Reaction: SAE (see section 8.1.2) that is unexpected as defined in section 8.2 and possibly related to the investigational medicinal products.

8.2 Expected Adverse Events

Table 5. Summary of apremilast adverse reactions in psoriatic arthritis and/or psoriasis (Ref. SmPC section 4.8)

System organ class	Frequency	Adverse reaction
Infections and infestations	Common	Bronchitis
		Upper respiratory tract infection
		Nasopharyngitis
Immune system disorders	Uncommon	Hypersensitivity
Metabolism and nutrition disorders	Common	Decreased appetite
Psychiatric disorders	Common	Insomnia<br>Depression
	Uncommon	Suicidal ideation and behavior
Nervous system disorders	Common	Migraine
		Tension headache
		Headache
Respiratory, thoracic and mediastinal disorders	Common	Cough
Gastrointestinal disorders	Very common	Diarrhea
		Nausea
	Common	Vomiting
		Dyspepsia
		Frequent bowel movements
		Upper abdominal pain
		Gastroesophageal reflux disease

	Uncommon	Gastrointestinal hemorrhage
Skin and subcutaneous tissue disorders	Uncommon	Rash
Musculoskeletal and connective tissue disorders	Common	Back pain
General disorders and administrative site conditions	Common	Fatigue
Investigations	Uncommon	Weight decrease

For details refer to Investigator's brochure and SmPC. Expected AEs/SAEs according to the IMPs Summary of Product Characteristics (SmPC) will be recorded in the eCRF.

8.3 Abnormal Laboratory Values

An abnormal laboratory value is considered to be an AE if the abnormality:

- Results in discontinuation from the study;
- Requires treatment, modification/ interruption of IMP dose, or any other therapeutic intervention; or
- Is judged to be of significant clinical importance.

Regardless of severity grade, only laboratory abnormalities that fulfill a seriousness criterion need to be documented as a serious adverse event.

If a laboratory abnormality is one component of a diagnosis or syndrome, then only the diagnosis or syndrome should be recorded on the AE page of the CRF. If the abnormality was not a part of a diagnosis or syndrome, then the laboratory abnormality should be recorded as the AE. If possible, the laboratory abnormality should be recorded as a medical term and not simply as an abnormal laboratory result (e.g., record thrombocytopenia rather than decreased platelets).

8.4 Pregnancy

Pregnancies and suspected pregnancies (including elevated βhCG or positive pregnancy test in a female subject of childbearing potential regardless of age or disease state) occurring while the subject is on IMP, or within 28 days of the subject's last dose of IMP, are considered immediately reportable events. IMP is to be discontinued immediately. The pregnancy/ suspected pregnancy must be reported to the Sponsor immediately by email or phone and by sending a completed Pregnancy Report Form or approved equivalent form to the Sponsor to be recorded in the subject's Viedoc file.

The female subject may be referred to an obstetrician-gynecologist or another appropriate healthcare professional for further evaluation.

The investigators will follow the female subject until completion of the pregnancy, and must notify the Sponsor immediately about the outcome of the pregnancy (either normal or abnormal

outcome) by email or phone and by sending a completed Pregnancy Report Form or approved equivalent form to the Sponsor.

If the outcome of the pregnancy was abnormal (e.g., spontaneous or therapeutic abortion), the Investigator should report the abnormal outcome as an AE. If the abnormal outcome meets any of the serious criteria, it must be reported as an SAE to Sponsor within 24 hours of the Investigator's knowledge of the event using the SAE Report Form or approved equivalent form.

All neonatal deaths that occur within 28 days of birth should be reported, without regard to causality, as SAEs. In addition, any infant death after 28 days that the Investigator suspects is related to the in-utero exposure to the IMP should also be reported to Sponsor as an SAE within 24 hours of the Investigator's knowledge of the event using the SAE Report Form or approved equivalent form.

8.5 Recording of Adverse Events

If the patient has experienced adverse event(s), the Investigator will record the following information in the CRF:

The nature of the event(s) will be described by the Investigator in precise standard medical terminology (i.e. not necessarily the exact words used by the patient).

- **Duration:** For both AEs and SAEs, the Investigator will provide a record of the start and stop dates of the event.

- **Severity/ Intensity:** For both AEs and SAEs, the Investigator must assess the severity / intensity of the event

Mild

- Asymptomatic or mild symptoms; clinical or diagnostic observations only
- Intervention not indicated
- ADLs (Activities of Daily Life) minimally or not affected
- No or minimal intervention/therapy may be required

Moderate

- Symptom(s) cause moderate discomfort
- Local or noninvasive intervention indicated
- More than minimal interference with ADLs but able to carry out daily social and functional activities.
- Drug therapy may be required

Severe (could be non-serious or serious)

- Symptoms causing severe discomfort/pain
- Symptoms requiring medical/surgical attention/intervention
- Interference with ADLs including inability to perform daily social and functional activities (e.g., absenteeism and/or bed rest)
- Drug therapy is required

It is important to distinguish between serious and severe AEs. Severity is a measure of intensity whereas seriousness is defined by the criteria in Section 8.1. An AE of severe intensity need not necessarily be considered serious. For example, nausea that persists for several hours may be considered severe nausea but is not an SAE. On the other hand, a stroke that results in only a limited degree of disability may be considered a mild stroke but would be an SAE.

- **Causality:** The Investigator must determine the relationship between the administration of IMP and the occurrence of an AE/SAE as Not Suspected or Suspected as defined below:

Not suspected:	A causal relationship of the adverse event to IMP administration is **unlikely or remote**, or other medications, therapeutic interventions, or underlying conditions provide a sufficient explanation for the observed event.
Suspected:	There is a **reasonable possibility** that the administration of IMP caused the adverse event. 'Reasonable possibility' means there is evidence to suggest a causal relationship between the IP and the adverse event.

Causality should be assessed and provided for every AE/SAE based on currently available information. Causality is to be reassessed and provided as additional information becomes available.

- **Action taken:** The Investigator will report the action taken with IMP as a result of an AE or SAE, as applicable (e.g., discontinuation or reduction of IP, as appropriate) and report if concomitant and/or additional treatments were given for the event.
- **Outcome** of the adverse event – whether the event is resolved or still ongoing.

8.6 Reporting Procedure

8.6.1 AEs and SAEs

All adverse events and serious adverse events that should be reported as defined in section 8.1.1 will be recorded in the patient's CRF.

SAEs must be reported to the Sponsor within 24 hours after the Investigator(s) has gained knowledge of the SAE. Every SAE must be documented by the Investigator in Viedoc as part of the CRF. The Serious Adverse Event Report Form must be completed, signed and sent to the sponsor. The initial report shall promptly be followed by detailed, written reports if necessary. The initial and follow-up reports shall identify the trial subjects by unique code numbers assigned to the latter.

The Sponsor keeps detailed records of all SAEs reported by the Investigators and performs an evaluation with respect to causality and expectedness. Based on, among other, SAE reports the Sponsor will evaluate whether the risk/benefit ratio associated with study is changed.

8.6.2 SUSARs

SUSARs will be reported to the Competent Authority (SLV) according to national regulation. The following timelines should be followed:

The Sponsor will ensure that all relevant information about suspected serious unexpected adverse reactions that are fatal or life-threatening is recorded and reported as soon as possible to the Competent Authority in any case no later than seven (7) days after knowledge by the Sponsor of such a case, and that relevant follow-up information is subsequently communicated within an additional eight (8) days.

The Sponsor will report all other suspected serious unexpected adverse reactions to the Competent Authority concerned as soon as possible but within a maximum of fifteen (15) days of first knowledge.

SUSARs will be reported using the CIOMS form since OUH is not connected to EudraVigilance.

8.6.3 Annual Safety Report

Once a year throughout the clinical trial, the Sponsor will provide the Competent Authority (SLV) with an annual safety report. The format will comply with national requirements.

8.6.4 Clinical Study Report

The adverse events and serious adverse events occurring during the study will be discussed in the safety evaluation part of the Clinical Study Report.

8.7 Procedures in Case of Emergency

The Investigator is responsible for assuring that there are procedures and expertise available to cope with emergencies during the study.

Randomization codes will be provided by Celgene (from May 1, 2020 Amgen) and available at CTU. Emergency unblinding of a patient's treatment allocation will be possible in Viedoc.

In the event of SUSARs, every effort should be made to contact the medical officer or investigators before revealing a patient’s allocated treatment during the study, to avoid unblinding that is not strictly necessary for the medical care of the patient.

9 DATA MANAGEMENT AND MONITORING

9.1 Case Report Forms

The Clinical Data Management System (CDMS) used for the eCRF in this study is Viedoc. The setup of the study specific eCRF in the CDMS will be performed by CTU, OUH. The eCRF system will be FDA Code of Federal Regulations 21 Part 11 compliant.

The designated investigator staff will enter the data required by the protocol into the eCase report forms (eCRF). The Investigator is responsible for assuring that data entered into the eCRF is complete, accurate, and that entry is performed in a timely manner. The signature of the Investigator will attest the accuracy of the data on each eCRF. If any assessments are omitted, the reason for such omissions will be noted on the eCRFs. Corrections, with the reason for the corrections will also be recorded.

After database lock, the Investigator will receive a digital copy of the subject data for archiving at the investigational site.

9.2 Source Data

Source data are all information in original records and certified copies of original records of clinical findings, observations, or other activities in a clinical trial necessary for the reconstruction and evaluation of the trial. Source data are contained in source documents (original records or certified copies).

The medical records for each patient should contain information that is important for the patient's safety and continued care, and to fulfill the requirement that critical study data should be verifiable.

To achieve this, the medical records of each patient should clearly describe at least:

- That the patient is participating in the study, e.g. by including the enrollment number and the study code or other study identification;
- Date when Informed Consent was obtained from the patient and statement that patient received a copy of the signed and dated Informed Consent;
- Results of all assessments confirming a patient's eligibility for the study;
- Diseases (past and current; both the disease studied and others, as relevant);
- Surgical history, as relevant;
- Treatments withdrawn/withheld due to participation in the study;
- Results of assessments performed during the study;
- Treatments given, changes in treatments during the study and the time points for the changes;
- Visits to the clinic / telephone contacts during the study, including those for study purposes only;
- Non-Serious Adverse Events and Serious Adverse Events (if any) including causality assessments;
- Date of, and reason for, discontinuation from study treatment;
- Date of, and reason for, withdrawal from study;
- Date of death and cause of death, if available;
- Additional information according to local regulations and practice.

9.3 Study Monitoring

The investigator will be visited on a regular basis by the Clinical Study Monitor, who will check the following:

- Informed consent process
- Reporting of adverse events and all other safety data

- Adherence to protocol
- Maintenance of required regulatory documents
- Study supply accountability
- Facilities and equipment (example: laboratory, pharmacy, ECG machine, etc.) if applicable
- Data completion on the CRFs including source data verification (SDV).

The monitor will review the relevant CRFs for accuracy and completeness and will ask the site staff to adjust any discrepancies as required.

Sponsor's representatives (e.g. monitors, auditors) and/or competent authorities will be allowed access to source data for source data verification in which case a review of those parts of the hospital records relevant to the study will be required.

9.4 Confidentiality

The Investigator shall arrange for the secure retention of the patient identification and the code list. Patient files shall be kept for the maximum period of time permitted by each hospital. The study documentation (CRFs, Site File etc.) shall be retained and stored during the study and for 15 years after study closure. All information concerning the study will be stored in a safe place inaccessible to unauthorized personnel.

9.5 Database management

The Clinical Trial Unit, OUH, will perform data management. The Data management procedures will be performed in accordance with the department's SOPs and ICH guidelines. The data management process will be described in the study specific data handling plan and the study specific data handling report after database closure.

Data entered into the eCRF will be validated as defined in the data validation plan. Validation includes, but is not limited to, validity checks (e.g. range checks), consistency checks and customized checks (logical checks between variables to ensure that study data are accurately reported) for eCRF data and external data (e.g. laboratory data). A majority of edit checks will be triggered during data entry and will therefore facilitate efficient 'point of entry' data cleaning.

Data management personnel will perform both manual eCRF review and review of additional electronic edit checks to ensure that the data are complete, consistent and reasonable. The electronic edit checks will run continually throughout the course of the study and the issues will be reviewed manually online to determine what action needs to be taken.

Manual queries may be added to the system by clinical data management or study monitor. Clinical data managers and study monitors are able to remotely and proactively monitor the patient eCRFs to improve data quality.

All updates to queried data will be made by authorized study center personnel only and all modifications to the database will be recorded in an audit trail. Once the queries have been resolved, eCRFs will be signed by electronic signature. Any changes to signed eCRFs will be approved and resigned by the Investigator.

Once the full set of eCRFs have been completed and locked, the Sponsor will authorize database lock and all electronic data will be sent to the designated statistician for analysis. Subsequent changes to the database will then be made only by written agreement.

The data will be stored in a dedicated and secured area at OUH. Data will be stored in a de-identified manner, where each study participant is recognizable by his/her unique trial subject number. The data will be stored until 15 years after completion of the study.

10 STATISTICAL METHODS AND DATA ANALYSIS

10.1 Determination of Sample Size

Helgesen et al.(10) found in a randomized trial a comparable effect of photodynamic therapy and topical corticosteroid, with mean GELP score reduced from 11 (standard deviation 4.5) at baseline to 7 (standard deviation 4.2) after 24 weeks follow-up. We assume a similar mean difference and standard deviation in GELP score at week 16 between apremilast-treated patients and placebo-treated patients as between the baseline and 24 weeks measurements in the previous trial reported by Helgesen et al.

To obtain 80% statistical power from an independent samples t-test with 5% significance level, mean difference of 4 and standard deviations in the two groups of 4.5 and 4.2, 20 subjects are required in each group. Based on this sample size calculation, and to account for a small drop our rate, we want to include 21 patients in both the apremilast and the placebo-treated group, i.e. 42 patients included in total.

10.2 Randomization

10.2.1 Allocation- sequence generation

The allocation-sequence will be done by permuted block randomization with unequal random block sizes and a 1:1 ratio of allocation (i.e. patients are allocated with equal probabilities to treatments) using appropriate statistical statistical software. A person not involved in the trial will generate the allocation-sequence.

Details of block size and allocation sequence will be provided in a separate document unavailable to study personnel enrolling patients and assigning treatment.

10.2.2 Allocation- procedure to randomize a patient

Allocation will be done in Viedoc. The allocation list will be stored at the Clinical Trial Unit, OUH, and not be available to the clinical investigators.

The study site will be provided with batches of treatment kits from Celgene (from May 1, 2020 Amgen) on a regular basis. A specific kit number identifies each treatment kit. When a patient is deemed eligible and ready for randomization, the Investigator will receive the patient's kit number through the eCRF system upon randomization. A treatment kit with the corresponding number is then given to the patient.

10.2.1 Blinding and emergency unblinding

Investigators, patients and statistician will be blinded for treatment allocation up to week 24. Unblinding of the treatment allocation is permissible only if the safety and well-being of the patient is being compromised. The decision to reveal the treatment allocation during the study may only be done by one of the Investigators. Treatment allocation will be available through the eCRF system and only to the Investigators, with a clear statement that unblinding must be done for emergency reasons only and in compliance with the protocol. The date and time of un-blinding must be documented in the eCRF and in the patient's hospital records.

In the event of an SAE, the investigator may only break the treatment code if the appropriate future management of the patient necessitates knowledge of the current treatment. Although it is advantageous to retain the blind for all patients prior to final trial analysis, when an SAE may be a serious adverse reaction unexpected or otherwise judged reportable on an expedited basis, it is recommended that the blind should be broken only for that specific patient, by the Sponsor or its designee, even if the investigator has not broken the blinding.

10.3 Population for Analysis

The following population will be considered for the analyses:

Intention to treat (ITT) population: All randomized participants, regardless of protocol adherence.

In addition, per-protocol analyses will be conducted.

10.4 Planned analyses

The main statistical analysis is planned when

- The planned number of patients have been included
- All included patients have either finalized their week 24 assessment or has/is withdrawn according to protocol procedures
- All data have been entered, verified and validated according to the data management plan

Prior to the main statistical analysis, the database will be locked for further entering or altering of data. A separate statistical analysis plan (SAP) will provide further details on the planned statistical analyses. The SAP will be finalized, signed and dated prior to database lock. The treatment allocation will be revealed after the database lock and used in the statistical analysis. Deviation from the original statistical plan will be described and justified in the Clinical Study Report. Amendments to plan can be done until day of database lock

10.5 Statistical Analysis

10.5.1 Statistical Hypothesis

This protocol is designed to establish the superiority of apremilast compared to placebo treatment on disease activity measured by GELP score after 24 weeks treatment in patients with moderate to severe female genital erosive lichen planus.

The primary null hypothesis is that there is no difference between the treatment groups in GELP score after 24 weeks treatment.

The primary alternative hypothesis is that there is a difference between the treatment groups in GELP score after 24 weeks treatment.

There is only one identified primary hypothesis test in this trial. All other efficacy analyses will be regarded as supportive or exploratory.

10.5.2 Statistical Decision Rule

This protocol is designed to address a single primary hypothesis. Superiority of apremilast is claimed if the null hypothesis is rejected on the 5% significance level, and the estimated treatment difference is in favour of aprimelast.

10.5.3 Primary analysis

The primary outcome, i.e. mean difference in GELP score at 24 weeks between the randomized groups, will be assessed with an ANCOVA model adjusting for the GELP score at baseline. We will use a 5% significance level in statistical analysis. The result will be reported as mean difference between the apremilast and placebo treated patients with a 95% confidence interval and a p-value as estimated from the ANCOVA model.

10.5.4 Secondary analyses

Continuous secondary outcomes at 4-, 16- and 24-weeks follow-up will be assessed using the same statistical principle as for the primary outcome, i.e. an ANCOVA model adjusting for the measured outcome at baseline. Categorical outcomes will be assessed with Pearson chi-square tests or logistic regression models as appropriate. In addition, statistical analysis using mixed models for repeated measurements will be conducted.

10.5.5 Descriptive statistics

Demographic and baseline characteristics will be presented using mean, standard deviation, number of observations and percentages as appropriate.

10.5.6 Missing data and intercurrent events

If missing data is regarded as having a significant effect on the conclusions of the trial, sensitivity analyses with different methods for handling missing data will be included. Such methods may include complete case analyses, last observation carried forward, worst case/best case imputation and multiple imputation techniques. Statistical analysis with mixed models for repeated measurements will also be conducted to obtain robust estimates in case of missing data.

Other intercurrent events, such as the use of rescue topical steroid crème, might also influence the conclusion of the trial.

The precise handling of missing data and other intercurrent events affecting the efficacy interpretation of the trial results will be detailed in the statistical analysis plan.

11 STUDY MANAGEMENT

11.1 Investigator Delegation Procedure

The Principal Investigator is responsible for making and updating a “delegation of tasks” listing all the involved co-workers and their role in the project. She will ensure that appropriate training relevant to the study is given to all of these staff, and that any new information of relevance to the performance of this study is forwarded to the staff involved.

11.2 Protocol Adherence

Investigators ascertain they will apply due diligence to avoid protocol deviations.
All significant protocol deviations will be recorded and reported in the Clinical Study Report (CSR).

11.3 Study Amendments

If it is necessary for the study protocol to be amended, the amendment and/or a new version of the study protocol (Amended Protocol) must be notified to and approved by the Competent Authority and the Ethics Committee according to EU and national regulations.

11.4 Audit and Inspections

Authorized representatives of a Competent Authority and Ethics Committee may visit the center to perform inspections, including source data verification. Likewise, the representatives from the Sponsor may visit the center to perform an audit. The purpose of an audit or inspection is to systematically and independently examine all study-related activities and documents to determine whether these activities were conducted, and data were recorded, analyzed, and accurately reported according to the protocol, Good Clinical Practice (ICH GCP), and any applicable regulatory requirements. The Principal Investigator will ensure that the inspectors and auditors will be provided with access to source data/documents.

12 ETHICAL AND REGULATORY REQUIREMENTS

The study will be conducted in accordance with ethical principles that have their origin in the Declaration of Helsinki and are consistent with ICH/Good Clinical Practice and applicable regulatory requirements. Registration of patient data will be carried out in accordance with national personal data laws.

12.1 Ethics Committee Approval

The study protocol, including the patient information and informed consent form to be used, must be approved by the regional ethics committee before enrolment of any patients into the study.

The Investigator is responsible for informing the ethics committee of any serious and unexpected adverse events and/or major amendments to the protocol as per national requirements.

12.2 Other Regulatory Approvals

The protocol will be submitted and approved by the applicable competent authorities before commencement of the study.

The study will also be registered in www.clinicaltrials.gov before inclusion of the first patient.

12.3 Informed Consent Procedure

The Investigator is responsible for giving the patients full and adequate verbal and written information about the nature, purpose, possible risk and benefit of the study. They will be informed as to the strict confidentiality of their patient data, but that their medical records may be reviewed for trial purposes by authorized individuals other than their treating physician.

It will be emphasized that the participation is voluntary and that the patient is allowed to refuse further participation in the protocol whenever she wants. This will not prejudice the patient's subsequent care. Documented informed consent must be obtained for all patients included in the study before they are registered in the study. This will be done in accordance with the national and local regulatory requirements. The Investigator is responsible for obtaining signed informed consent.

A copy of the patient information and consent will be given to the patients. The signed and dated patient consent forms will be filed in the Investigator Site File binder and also scanned to be part of the patient's electronic medical record at the hospital.

12.4 Subject Identification

The Investigator is responsible for keeping a list of all patients (who have received study treatment or undergone any study specific procedure) including patient's date of birth and personal number, full names and last known addresses.

The patients will be identified in the CRFs by patient number, initials and date of birth.

13 TRIAL SPONSORSHIP AND FINANCING

Sponsor of the study is Oslo University Hospital. The study is an Investigator Initiated Trial supported by funds from pharmaceutical Company Celgene (manufacturer of apremilast).

Celgene will cover the following expenses (total 777 867 NOK)

- Study medication (apremilast and placebo)
- Monitoring and data collection/ CRF (by the Clinical Trial Unit, OUH): 136,695 NOK
- Clinical visits/phone interviews/pharmacy/ travel expenses (patients): 561,172 NOK
- Biopsies/immunohistochemical analyses: 80 000 NOK

Details on the cooperation with Celgene are found in the Agreement between Oslo University Hospital and Celgene International.
Following Celgene's sale to Amgen of the global rights to Otezla®, rights and obligations under the contract with Celgene is transferred to Amgen fra May 1, 2020. Details of this transfer is found in the Novation and Amendment Agreement.

14 TRIAL INSURANCE

The Principal Investigator has insurance coverage for this study through membership of the Drug Liability Association (see http://www.laf.no for more details).

15 PUBLICATION POLICY

Upon study completion and finalization, the results of the study will be submitted for publication in a peer-reviewed scientific medical journal and/or posted in a publicly accessible database of clinical studies.

The results of this study will also be submitted to the Competent Authority and the Ethics Committee according to EU and national regulations.

All personnel who have contributed significantly with the planning and performance of the study (Vancouver convention 1988) may be included in the list of authors.

The Principal Investigator (ALH) and investigator (KHS) will have the right to publish the report regardless of outcomes. The Sponsor (OUS) and Celgene (from May 1, 2020 Amgen) will be involved in modification(s) of the protocol.

16 LIST OF APPENDICES

A Blinded Kit Template

APPENDIX A -BLINDED KIT TEMPLATE

Blinded Titration Card – 10-20-30mg

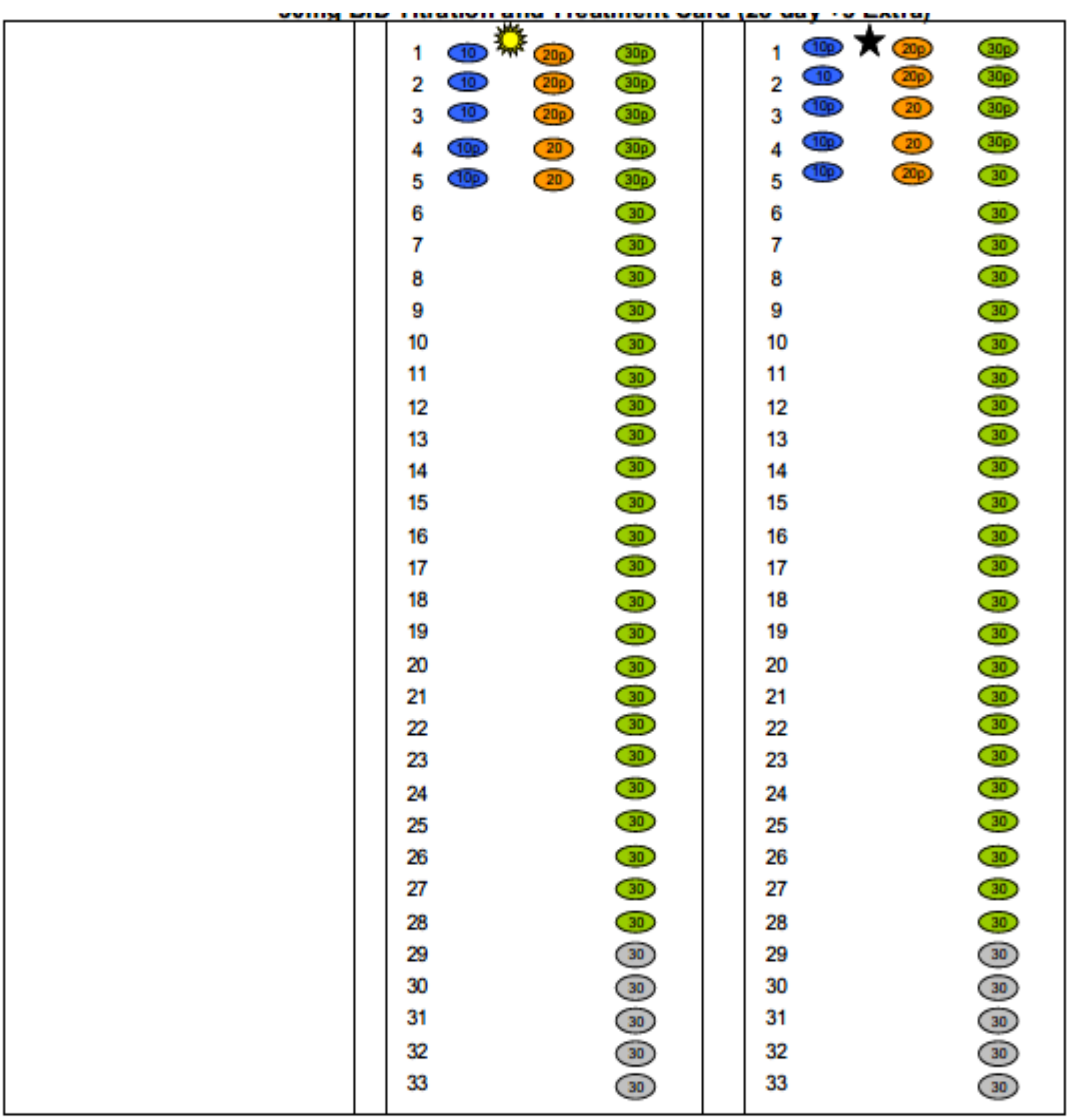

[illegible]

1	10	20p	30p	1	10p	20p	30p
2	10	20p	30p	2	10	20p	30p
3	10	20p	30p	3	10p	20	30p
4	10p	20	30p	4	10p	20	30p
5	10p	20	30p	5	10p	20p	30
6			30	6			30
7			30	7			30
8			30	8			30
9			30	9			30
10			30	10			30
11			30	11			30
12			30	12			30
13			30	13			30
14			30	14			30
15			30	15			30
16			30	16			30
17			30	17			30
18			30	18			30
19			30	19			30
20			30	20			30
21			30	21			30
22			30	22			30
23			30	23			30
24			30	24			30
25			30	25			30
26			30	26			30
27			30	27			30
28			30	28			30
29			30	29			30
30			30	30			30
31			30	31			30
32			30	32			30
33			30	33			30

30mg – Treatment Card (Active)

Note: To support ongoing blinding across titration & treatment patients will need to take 3 tablets a day for the first 5 days with each new card received by the patient.

1	10p	20p	30	1	10p	20p	30
2	10p	20p	30	2	10p	20p	30
3	10p	20p	30	3	10p	20p	30
4	10p	20p	30	4	10p	20p	30
5	10p	20p	30	5	10p	20p	30
6			30	6			30
7			30	7			30
8			30	8			30
9			30	9			30
10			30	10			30
11			30	11			30
12			30	12			30
13			30	13			30
14			30	14			30
15			30	15			30
16			30	16			30
17			30	17			30
18			30	18			30
19			30	19			30
20			30	20			30
21			30	21			30
22			30	22			30
23			30	23			30
24			30	24			30
25			30	25			30
26			30	26			30
27			30	27			30
28			30	28			30
29			30	29			30
30			30	30			30
31			30	31			30
32			30	32			30
33			30	33			30

Blinded Titration/Treatment Card - PBO

[illegible]

1	10p	20p	30p	1	10p	20p	30p
2	10p	20p	30p	2	10p	20p	30p
3	10p	20p	30p	3	10p	20p	30p
4	10p	20p	30p	4	10p	20p	30p
5	10p	20p	30p	5	10p	20p	30p
6			30p	6			30p
7			30p	7			30p
8			30p	8			30p
9			30p	9			30p
10			30p	10			30p
11			30p	11			30p
12			30p	12			30p
13			30p	13			30p
14			30p	14			30p
15			30p	15			30p
16			30p	16			30p
17			30p	17			30p
18			30p	18			30p
19			30p	19			30p
20			30p	20			30p
21			30p	21			30p
22			30p	22			30p
23			30p	23			30p
24			30p	24			30p
25			30p	25			30p
26			30p	26			30p
27			30p	27			30p
28			30p	28			30p
29			30p	29			30p
30			30p	30			30p
31			30p	31			30p
32			30p	32			30p
33			30p	33			30p

17 REFERENCES

1. Lewis FM, Bogliatto F. Erosive vulval lichen planus--a diagnosis not to be missed: a clinical review. Eur J Obstet Gynecol Reprod Biol. 2013;171(2):214-9.
2. Cooper SM, Wojnarowska F. Influence of treatment of erosive lichen planus of the vulva on its prognosis. Arch Dermatol. 2006;142(3):289-94.
3. Helgesen AL, Gjersvik P, Jebsen P, Kirschner R, Tanbo T. Vaginal involvement in genital erosive lichen planus. Acta Obstet Gynecol Scand. 2010;89(7):966-70.
4. Simpson RC, Thomas KS, Leighton P, Murphy R. Diagnostic criteria for erosive lichen planus affecting the vulva: an international electronic-Delphi consensus exercise. Br J Dermatol. 2013;169(2):337-43.
5. Cooper SM, Dean D, Allen J, Kirtschig G, Wojnarowska F. Erosive lichen planus of the vulva: weak circulating basement membrane zone antibodies are present. Clin Exp Dermatol. 2005;30(5):551-6.
6. Moyal-Barracco M, Edwards L. Diagnosis and therapy of anogenital lichen planus. Dermatol Ther. 2004;17(1):38-46.
7. Cribier B, Frances C, Chosidow O. Treatment of lichen planus. An evidence-based medicine analysis of efficacy. Arch Dermatol. 1998;134(12):1521-30.
8. Ho JK, Hantash BM. Systematic review of current systemic treatment options for erosive lichen planus. Expert Review of Dermatology. 2012;7(3):269-82.
9. Cheng S, Kirtschig G, Cooper S, Thornhill M, Leonardi-Bee J, Murphy R. Interventions for erosive lichen planus affecting mucosal sites. The Cochrane database of systematic reviews. 2012(2):CD008092.
10. Helgesen AL, Warloe T, Pripp AH, Kirschner R, Peng Q, Tanbo T, et al. Vulvovaginal photodynamic therapy vs. topical corticosteroids in genital erosive lichen planus: a randomized controlled trial. Br J Dermatol. 2015;173(5):1156-62.
11. Kumar N, Goldminz AM, Kim N, Gottlieb AB. Phosphodiesterase 4-targeted treatments for autoimmune diseases. BMC Med. 2013;11:96.
12. Hatemi G, Melikoglu M, Tunc R, Korkmaz C, Turgut Ozturk B, Mat C, et al. Apremilast for Behcet's syndrome--a phase 2, placebo-controlled study. N Engl J Med. 2015;372(16):1510-8.
13. Schafer PH, Truzzi F, Parton A, Wu L, Kosek J, Zhang L-H, et al. Phosphodiesterase 4 in inflammatory diseases: Effects of apremilast in psoriatic blood and in dermal myofibroblasts through the PDE4/CD271 complex. Cell Signal. 2016;28(7):753-63.
14. Perez-Aso M, Montesinos MC, Mediero A, Wilder T, Schafer PH, Cronstein B. Apremilast, a novel phosphodiesterase 4 (PDE4) inhibitor, regulates inflammation through multiple cAMP downstream effectors. Arthritis Res Ther. 2015;17:249.
15. Samrao A, Berry TM, Goreshi R, Simpson EL. A pilot study of an oral phosphodiesterase inhibitor (apremilast) for atopic dermatitis in adults. Arch Dermatol. 2012;148(8):890-7.
16. Strand V, Fiorentino D, Hu C, Day RM, Stevens RM, Papp KA. Improvements in patient-reported outcomes with apremilast, an oral phosphodiesterase 4 inhibitor, in the treatment of moderate to severe psoriasis: results from a phase IIb randomized, controlled study. Health and quality of life outcomes. 2013;11:82.
17. ClinicalTrials.gov. USA: National Institutes of Health [cited 2017 24.04.]. Available from: https://clinicaltrials.gov/ct2/results?term=apremilast&cond=%22Skin+Diseases%22&pg=2.
18. Paul J, Foss CE, Hirano SA, Cunningham TD, Pariser DM. An open-label pilot study of apremilast for the treatment of moderate to severe lichen planus: a case series. J Am Acad Dermatol. 2013;68(2):255-61.
19. Bettencourt M. Oral Lichen Planus Treated With Apremilast. Journal of drugs in dermatology : JDD. 2016;15(8):1026-8.
20. Hafner J, Gubler C, Kaufmann K, Nobbe S, Navarini AA, French LE. Apremilast Is Effective in Lichen Planus Mucosae-Associated Stenotic Esophagitis. Case Rep Dermatol. 2016;8(2):224-6.
21. AbuHilal M, Walsh S, Shear N. Treatment of recalcitrant erosive oral lichen planus and desquamative gingivitis with oral apremilast. J Dermatol Case Rep. 2016;10(3):56-7.
22. Crowley J, Thaci D, Joly P, Peris K, Papp KA, Goncalves J, et al. Long-term safety and tolerability of apremilast in patients with psoriasis: Pooled safety analysis for >/=156 weeks from 2 phase 3, randomized, controlled trials (ESTEEM 1 and 2). J Am Acad Dermatol. 2017.
23. Paul C, Cather J, Gooderham M, Poulin Y, Mrowietz U, Ferrandiz C, et al. Efficacy and safety of apremilast, an oral phosphodiesterase 4 inhibitor, in patients with moderate-to-severe plaque psoriasis over 52 weeks: a phase III, randomized controlled trial (ESTEEM 2). Br J Dermatol. 2015;173(6):1387-99.
24. Otezla prescribing information. www.celgene.com/content/uploads/otezla-pi.pdf: Celgene; revised 12/2015.
25. Zerilli T, Ocheretyaner E. Apremilast (Otezla): A New Oral Treatment for Adults With Psoriasis and Psoriatic Arthritis. P & T : a peer-reviewed journal for formulary management. 2015;40(8):495-500.
26. Celgene. Celgene Norway (personal communication 24.04.17)

27. (CIOMS) CfIOoMS. International Ethical Guidelines for Health-related Research Involving Humans. Fourth ed. Geneva, Switzerland: Council for International Organizations of Medical Sciences (CIOMS); 2016.
28. Papp K, Reich K, Leonardi CL, Kircik L, Chimenti S, Langley RG, et al. Apremilast, an oral phosphodiesterase 4 (PDE4) inhibitor, in patients with moderate to severe plaque psoriasis: Results of a phase III, randomized, controlled trial (Efficacy and Safety Trial Evaluating the Effects of Apremilast in Psoriasis [ESTEEM] 1). J Am Acad Dermatol. 2015;73(1):37-49.
29. Agency EM. Summary of Product Characteristics [cited 2018 31.01.]. Available from: https://ec.europa.eu/health/documents/community-register/2015/20150115130395/anx_130395_en.pdf.
30. Pascoe VL, Enamandram M, Corey KC, et al. Using the physician global assessment in a clinical setting to measure and track patient outcomes. JAMA dermatology. 2015;151(4):375-81.
31. Lundqvist EN, Wahlin YB, Bergdahl M, Bergdahl J. Psychological health in patients with genital and oral erosive lichen planus. J Eur Acad Dermatol Venereol. 2006;20(6):661-6.
32. Cheng H, Oakley A, Conaglen JV, Conaglen HM. Quality of Life and Sexual Distress in Women With Erosive Vulvovaginal Lichen Planus. J Low Genit Tract Dis. 2017;21(2):145-9.
33. Finlay AY, Khan GK. Dermatology Life Quality Index (DLQI)--a simple practical measure for routine clinical use. Clin Exp Dermatol. 1994;19(3):210-6.
34. Derogatis LR, Rosen R, Leiblum S, Burnett A, Heiman J. The Female Sexual Distress Scale (FSDS): initial validation of a standardized scale for assessment of sexually related personal distress in women. J Sex Marital Ther. 2002;28(4):317-30.
35. al. GDe. Manual of the General Health Questionnaire. Windsor, England: NFER Publishing; 1978.
36. Heads of Medicines Agencies: Clinical Trial Facilitation Group. Recommendations related to contraception and pregnancy testing in clinical trials. 2014. Available from: www.hma.eu.
